# Evolutionarily Repurposed Networks Reveal the Well-Known Antifungal Drug Thiabendazole to Be a Novel Vascular Disrupting Agent

**DOI:** 10.1371/journal.pbio.1001379

**Published:** 2012-08-21

**Authors:** Hye Ji Cha, Michelle Byrom, Paul E. Mead, Andrew D. Ellington, John B. Wallingford, Edward M. Marcotte

**Affiliations:** 1 Center for Systems and Synthetic Biology, Institute for Cellular and Molecular Biology, University of Texas at Austin, Austin, Texas, United States of America; 2 Department of Pathology, St. Jude Children's Research Hospital, Memphis, Tennessee, United States of America; 3 Department of Chemistry and Biochemistry, University of Texas at Austin, Austin, Texas, United States of America; 4 Howard Hughes Medical Institute & Section of Molecular Cell and Developmental Biology, University of Texas at Austin, Austin, Texas, United States of America; Stanford University, United States of Amreica

## Abstract

Studies in diverse organisms have revealed a surprising depth to the evolutionary conservation of genetic modules. For example, a systematic analysis of such conserved modules has recently shown that genes in yeast that maintain cell walls have been repurposed in vertebrates to regulate vein and artery growth. We reasoned that by analyzing this particular module, we might identify small molecules targeting the yeast pathway that also act as angiogenesis inhibitors suitable for chemotherapy. This insight led to the finding that thiabendazole, an orally available antifungal drug in clinical use for 40 years, also potently inhibits angiogenesis in animal models and in human cells. Moreover, in vivo time-lapse imaging revealed that thiabendazole reversibly disassembles newly established blood vessels, marking it as vascular disrupting agent (VDA) and thus as a potential complementary therapeutic for use in combination with current anti-angiogenic therapies. Importantly, we also show that thiabendazole slows tumor growth and decreases vascular density in preclinical fibrosarcoma xenografts. Thus, an exploration of the evolutionary repurposing of gene networks has led directly to the identification of a potential new therapeutic application for an inexpensive drug that is already approved for clinical use in humans.

## Abstract 2

Analysis of a genetic module repurposed between yeast and vertebrates reveals that a common antifungal medication is also a potent vascular disrupting agent.

## Author Summary

Yeast cells and vertebrate blood vessels would not seem to have much in common. However, we have discovered that during the course of evolution, a group of proteins whose function in yeast is to maintain cell walls has found an alternative use in vertebrates regulating angiogenesis. This remarkable repurposing of the proteins during evolution led us to hypothesize that, despite the different functions of the proteins in humans compared to yeast, drugs that modulated the yeast pathway might also modulate angiogenesis in humans and in animal models. One compound seemed a particularly promising candidate for this sort of approach: thiabendazole (TBZ), which has been in clinical use as a systemic antifungal and deworming treatment for 40 years. Gratifyingly, our study shows that TBZ is indeed able to act as a vascular disrupting agent and an angiogenesis inhibitor. Notably, TBZ also slowed tumor growth and decreased vascular density in human tumors grafted into mice. TBZ’s historical safety data and low cost make it an outstanding candidate for translation to clinical use as a complement to current anti-angiogenic strategies for the treatment of cancer. Our work demonstrates how model organisms from distant branches of the evolutionary tree can be exploited to arrive at a promising new drug.

## Introduction

Systems biology has shown great promise in providing a better understanding of human disease and in identifying new disease targets. These methods typically leave off once the target is identified, and further research transitions to established paradigms for drug discovery. However, the vast majority of molecular pathways that function in human disease are not specific to humans, but rather are conserved across vertebrates and even to very distantly related organisms. The remarkable growth of genetic data from tractable model organisms implies that most genetic modules relevant to human biology are currently best characterized in non-human species. Such evolutionary conservation, even when the homology of the systems to the human case is distant or perhaps non-obvious, should enable new drug design strategies.

Clearly, identification of deeply conserved gene networks in distant organisms opens the possibility of pursuing drug discovery in those organisms. While traditional methods of drug discovery focus on gene-by-gene rather than network- or system-level similarities, we suggest that phenologs—gene networks that while orthologous may nonetheless produce different phenotypes due to altered usage or organismal contexts [1]—can provide a basis not just for screening against a single protein, but also for simultaneous drug discovery efforts against multiple targets in parallel. Given the key roles that model organisms already play in biomedical research, identification of such deep homologies should also allow us to better leverage the particular strengths of the wide variety of animal models in order to rapidly test candidate drugs found from such an approach.

We recently developed a method for systematically discovering phenologs, and this approach identified a conserved module that is relevant to lovastatin sensitivity in yeast and is also responsible for regulating angiogenesis in vertebrates [1]. Angiogenesis, the process of forming new blood vessels, plays an essential role in development, reproduction, and tissue repair [2]. Because the vascular network supplies oxygen and nutrients to cancer cells as well as to normal cells, angiogenesis also governs the growth of many types of tumors, and is central to malignancy [2]–[5]. The vasculature is thus considered to be a major therapeutic target for drug development. Some cancers, such as the most common and deadly brain neoplasm, glioblastoma multiformae [6], are heavily vascularized, but have not responded to current angiogenesis inhibitors [7],[8]. Because new agents that target the vasculature would increase our arsenal for battling cancers resistant to current therapies [2]–[5], there is a clear clinical need for novel approaches to their identification.

**Figure 1:**
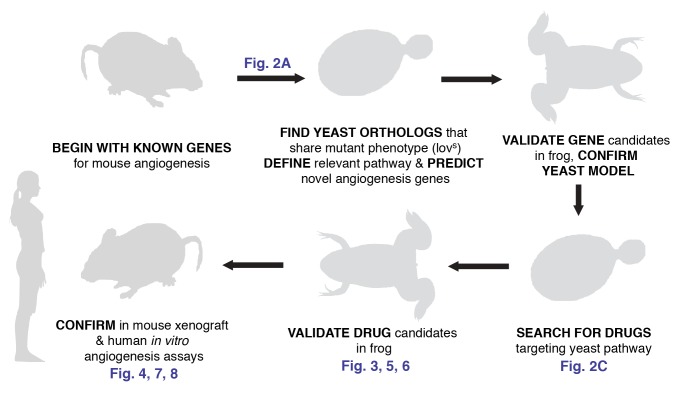
Overview of the evolutionary method used to discover a novel vascular disrupting agent. Strict reliance on the evolutionary conservation of the relevant gene module allowed for an experimental design exploiting the unique experimental advantages of each model organism, thus speeding the search for novel angiogenesis inhibitors. (Vector female silhouette under Creative Commons Attribution 2.0 from ‘Keep Fit’ Vector Pack, Blog.SpoonGraphics.)

Here, we have exploited data mining of genetic interactions in yeast, in vivo time-lapse imaging in a non-mammalian vertebrate, loss-of-function analysis in cultured human cells, and preclinical xenografts in mice to identify and characterize a novel anti-angiogenic small molecule (Figure 1). Excitingly, this compound is already FDA approved for use in treating certain infections in humans, making it an excellent candidate for rapid translation to the clinic. This research exemplifies a general strategy for exploiting deeply conserved genetic modules for drug screening, characterized by screens focused not on single genes but rather on conserved genetic modules and by a strong reliance on tractable model organisms in order to speed the discovery of therapeutics.

## Results and Discussion

### Identification of a Novel Small-Molecule Angiogenesis Inhibitor

**Figure 2:**
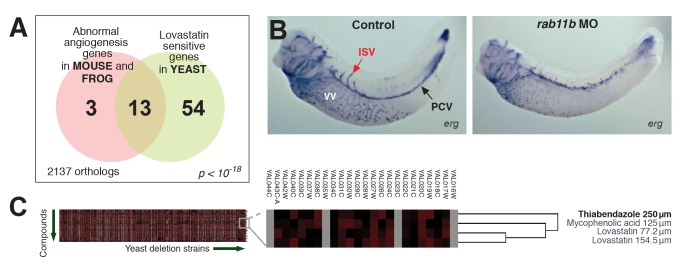
Identification of candidate angiogenesis inhibitors based upon genetic interactions with a yeast gene module. (A) Summary of the gene module (modified from [1]). Tests of genes associated with the yeast phenotype (lovastatin sensitivity) correctly identified novel angiogenesis genes, as in [1] and additionally shown in (B) for the gene *rab11b*. Morpholino (MO) knockdown of *rab11B* induces vascular defects in developing *Xenopus laevis* (frog) embryos, measured by in situ hybridization versus marker gene *erg*. ISV, intersomitic vein; PCV, posterior cardinal vein; VV, vitellin vein. (C) In an unbiased hierarchical clustering of compounds by their synthetic genetic interaction profiles with yeast genes (analyzing data from [13]), the action of TBZ is among those interacting with this gene module and also most similar to lovastatin, the signature compound affiliated with the angiogenesis gene module; hence, TBZ is a likely candidate angiogenesis inhibitor. Here, complete linkage clustering employing uncentered correlation coefficients is shown; additional clustering methods are illustrated in Figure S2.

The remarkable conservation of a genetic module that controls lovastatin sensitivity in yeast and angiogenesis in vertebrates ([1]; Figures 2AB and S1; Table S1) led us to test the possibility that small-molecule inhibitors modulating the yeast pathway might also act as angiogenesis inhibitors. Indeed, preliminary evidence suggests that lovastatin itself at least partly inhibits angiogenesis [9],[10] and may even reduce the incidence of melanoma [11],[12]. We therefore devised a strategy to exploit the evolutionary repurposing of this module in order to direct our search (Figure 1). Specifically, we desired to identify compounds in a manner that did not require their mechanism of action or even their biochemical target to match that of lovastatin; we thus employed a genetic strategy in yeast in order to select compounds that genetically interacted with this module. By computationally mining available large-scale chemical sensitivity datasets [13], candidate compounds were prioritized based upon their measured synthetic genetic interactions with yeast genes, using clustering algorithms to identify those compounds with genetic interaction profiles most similar to that of lovastatin (Figure 2C; Table S2; Figure S2). Notably, four out of eight prioritized chemicals were already known to modulate angiogenesis, indicating strong enrichment for angiogenesis effectors (Table S2).

One compound—thiabendazole (TBZ; 4-(1H-1,3-benzodiazol-2-yl)-1,3-thiazole)—stood out because it has already been approved by the U.S. Food and Drug Administration (FDA) for systemic oral use in humans (as an anti-fungal and anti-helminthic treatment). TBZ was initially marketed by Merck as Mintezol, and is now off-patent and issued as a generic under the trade names Apl-Luster, Mertect, Mycozol, Tecto, Tresaderm, and Arbotect. TBZ has been used by humans since its FDA approval in 1967, so its safety has been well-established. In animals, TBZ has no carcinogenic effects in either short- or long-term studies at doses up to 15 times the usual human dose [14],[15]. Moreover, TBZ does not appear to affect fertility in mice or rats, and it is not a mutagen in standard in vitro microbial mutagen tests, micronucleus tests, or host-mediated assays in vivo [14],[15]. Thus, TBZ was an outstanding candidate for further study.

### Thiabendazole Inhibits Angiogenesis

**Figure 3:**
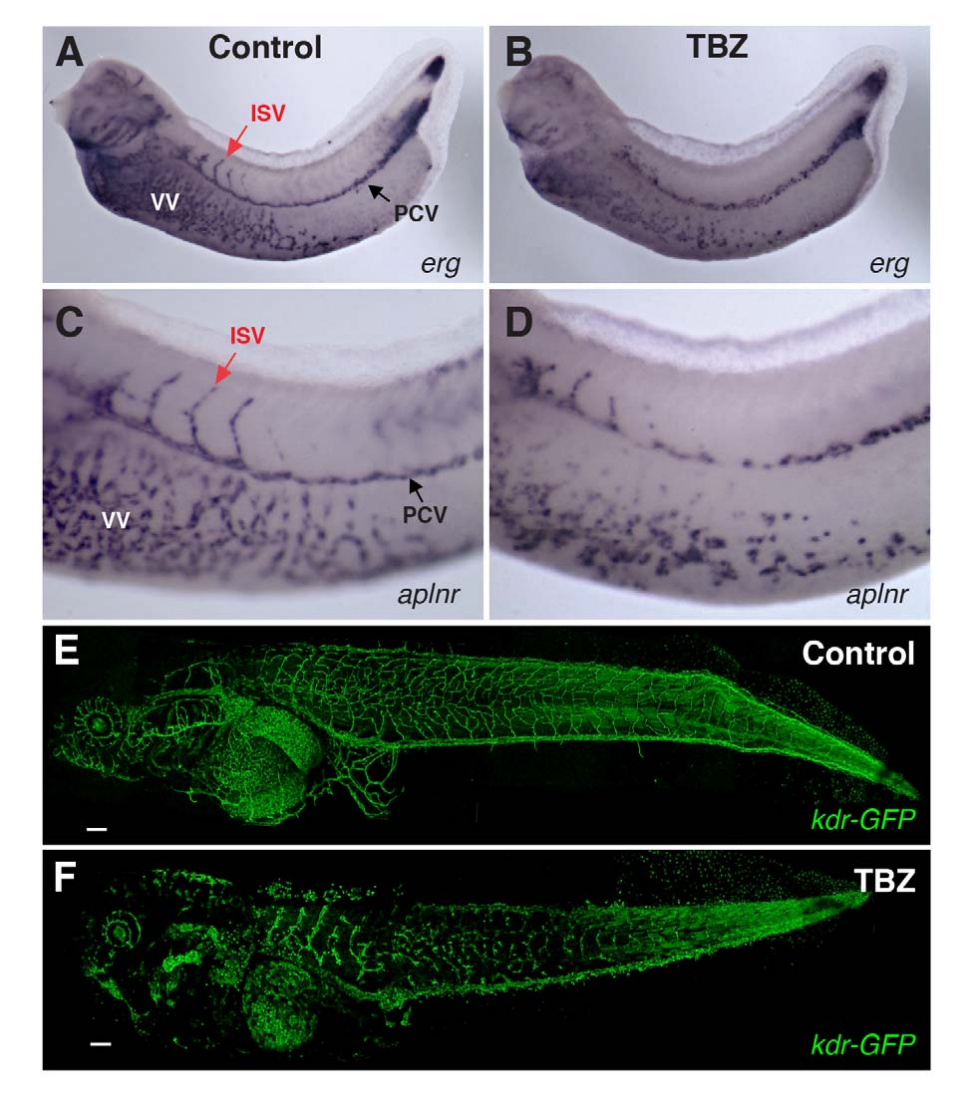
TBZ inhibits angiogenesis in vivo in *Xenopus* embryos. Formation of *Xenopus* embryo veins is disrupted, marked by expression of vascular reporter genes (A, B) *erg* and (C, D) *aplnr*, contrasting treatment with 1% DMSO control only (A, C) with 1% DMSO, 250 µM TBZ, treated at stage 31 and imaged at stages 35–36 (B, D). PCV, posterior cardinal vein; ISV, intersomitic vein; VV, vitellin veins. Similarly, TBZ disrupts vasculature imaged within a living *Xenopus* embryo and visualized by vascular specific GFP in *kdr:GFP* frogs from [19], contrasting the vasculature of stage 46 animals treated from stage 41 with the 1% DMSO control (E) or 1% DMSO, 250 µM TBZ (F). Scale bar, 200 µm.

**Figure 4:**
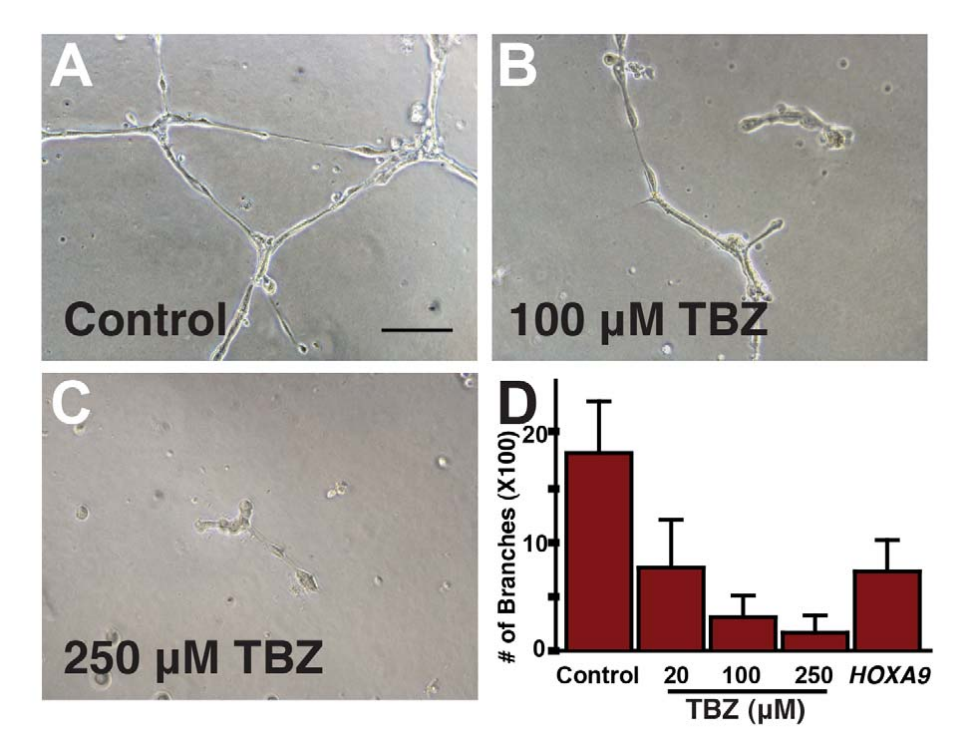
TBZ significantly disrupts tube formation in cultured human umbilical vein endothelial cells (HUVECs), an in vitro capillary model. Here, we show effects of 1% DMSO-treated control (A) versus 1% DMSO, 100 µM TBZ (B) and 1% DMSO, 250 µM TBZ (C). Scale bar, 100 µm. (D) Tube disruption is dose-dependent and comparable to that from silencing known pro-angiogenic gene *HOXA9*.

We first tested the effect of TBZ on the expression of vascular-specific genes in developing *Xenopus* embryos, which provide a rapid, tractable, and accurate model for in vivo studies of angiogenesis [16]–[19]. Using in situ hybridization to either the apelin-receptor (*aplnr*) or the vascular ETS factor (*erg*), we found that TBZ treatment severely impaired angiogenesis (Figure 3A–D). This result was confirmed in living embryos in which vasculature was visualized by expression of GFP under control of a *kdr* enhancer/promoter fragment (Figure 3E–F) [19]. Notably, TBZ also inhibited angiogenesis in a dose-dependent manner in cultured human endothelial cells (HUVECs), suggesting that the activity of TBZ is conserved in vertebrate*s* (Figure 4). We then sought to position the site of TBZ action relative to that of VEGF, as this growth factor is central to both normal and pathogenic angiogenesis [3],[4]. In frog embryos, ectopic VEGF potently induces ectopic angiogenesis [16], and this effect was blocked by TBZ, suggesting that the drug acts downstream of this key regulatory node (Figure S3).

These data implicate TBZ as an effective inhibitor of angiogenesis. Importantly, we observed angiogenesis inhibition in both human cells in vitro and in *Xenopus* embryos in vivo at a concentration of 100–250 µM. This dose corresponds to 20–50 mg/kg (Figures 3 and 4), which is notable because the oral LD50 of MINTEZOL is 1.3–3.6 g/kg, 3.1 g/kg, and 3.8 g/kg in the mouse, rat, and rabbit, respectively, and the human approved recommended maximum daily dose is 3 grams, corresponding to 50 mg/kg for 60 kg patients. Finally, we note that the overall morphology and patterning of TBZ-treated *Xenopus* embryos was grossly normal at the stages when the vasculature was severely disrupted (Figure S4). Consistent with this, TBZ has good safety data in humans and model animals at the doses for which we observe a specific inhibition of angiogenesis [14],[15].

We next asked if angiogenesis inhibition may be a general property of benzimidazoles. Examination of commercially available TBZ derivatives showed that this is not the case, with benzimidazole itself inactive at doses up to 1 mM and administration of other benzimidazoles causing diverse developmental defects but not angiogenesis inhibition (Figure S5). These findings are thus significant for demonstrating a high level of precision for this evolutionary approach to drug discovery.

### Thiabendazole Is a Vascular Disrupting Agent

We next sought to better understand the cellular basis for angiogenesis inhibition by TBZ. In the course of our studies, we noted an interesting feature of the vasculature in TBZ-treated embryos: disconnected and scattered arrays of cells in which vascular gene expression persisted (Figures 3B,D and S3). Hypothesizing that such morphological defects in the absence of changes to vascular gene expression may stem from direct impairment of vessel integrity, we tested the ability of TBZ to disrupt pre-existing vasculature by treatments at later stages, when blood vessels were already well formed and patent [20]. TBZ treatment elicited overt breakdown of established vasculature at these stages (Figure S6).

The ability of TBZ to disassemble extant blood vessels was especially significant because such an activity has recently drawn the attention of cancer biologists [4],[21],[22]. A new class of drugs called Vascular Disrupting Agents (VDAs) break down existing vascular structures, thereby disrupting blood flow, particularly within solid tumors [4],[21],[22]. No VDAs have as yet been approved for use in humans, although several such agents are therapeutically promising and are in phase II and III trials [4].

**Figure 5:**
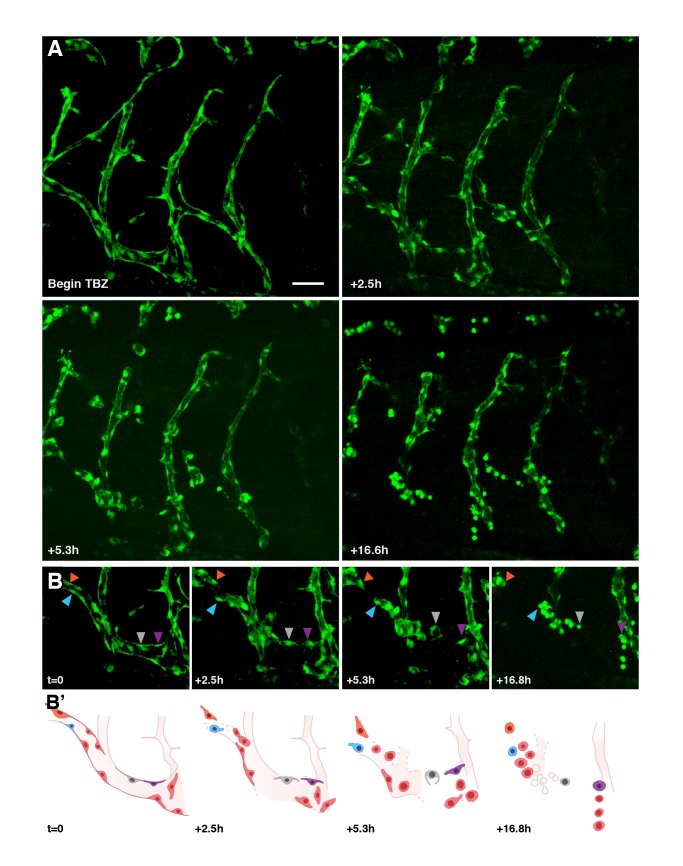
TBZ disrupts newly established vasculature, as visualized in vivo using time-lapse fluorescence microscopy within *kdr:GFP* frogs. Retraction and rounding of vascular endothelial cells (arrowheads) is apparent in TBZ-treated embryos (A, time lapse of frogs treated as in Figure 3) as compared with continued vascular growth in control animals (Figure S7). Scale bar, 80 µm. (B) A series with intermediate time points is shown for a sub-region of that shown in (A). (B′) Schematics of the images in (B) indicate positions of specific cells.

As a direct test of the vascular disrupting activity of TBZ, we performed time-lapse imaging of developing vasculature. Using *Kdr*-GFP transgenic embryos [19] and time-lapse confocal microscopy [23], we could effectively image developing vasculature in vivo for periods of up to 20 h. During this time, the growth of existing vessels and the sprouting of new vasculature could be easily followed (Figure S7; Movie S1). Treatment with TBZ completely prevented growth and sprouting of vessels, and moreover elicited a striking disintegration of established vessels after ∼90 min of exposure (Figures 5 and S8; Movie S2). Upon longer exposures, endothelial cells scattered and many underwent dramatic rounding (Figures 5A and S8; Movie S2).

These data demonstrate the efficacy of TBZ as a vascular disrupting agent. Previously defined VDAs can act either by targeting endothelial cells for selective cell death (e.g., ASA404 [24]) or by disrupting endothelial cell behaviors (e.g., combrestatin A4 [25]), and so we sought to distinguish between these two possible mechanisms for TBZ action. We noted that treatment with TBZ doses sufficient to severely perturb the vasculature elicited only modest increases in apoptosis in cultured HUVECs (Figure S9). Moreover, vascular gene expression in dispersed, rounded *kdr-GFP+* endothelial cells in vivo reliably persisted for up to 17 h after TBZ treatment (Figures 3F, 5, and S8).

**Figure 6:**
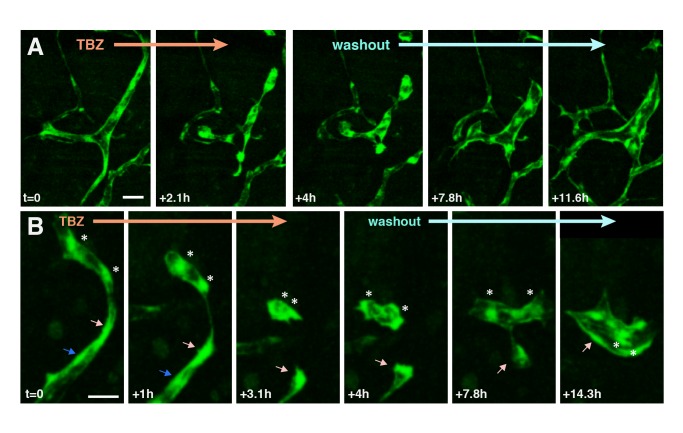
After disassembly of the vasculature by TBZ, washout of the drug leads to partial recovery of the vascular network. Two time series are shown in (A) and (B), imaged as in Figure 5. Following washout, cells dissociated by TBZ treatment recommence cell elongation and connection. Individual cells are indicated by arrows/asterisks. Scale bar in (A), 50 µm; in (B), 40 µm.

These data argue against a role for apoptosis in vascular disruption by TBZ. To test this idea more directly, we performed washout experiments. Compellingly, washout of the drug after overt TBZ-induced endothelial cell dispersal and rounding resulted in significant re-spreading of endothelial cells and re-formation of vessels in living *Xenopus* embryos assessed by time-lapse imaging (Figure 6; Movie S3). In several cases, widely separated *kdr-GFP*-positive endothelial cells reconnected into nascent vessels after washout of TBZ (Figure 6).

**Figure 7:**
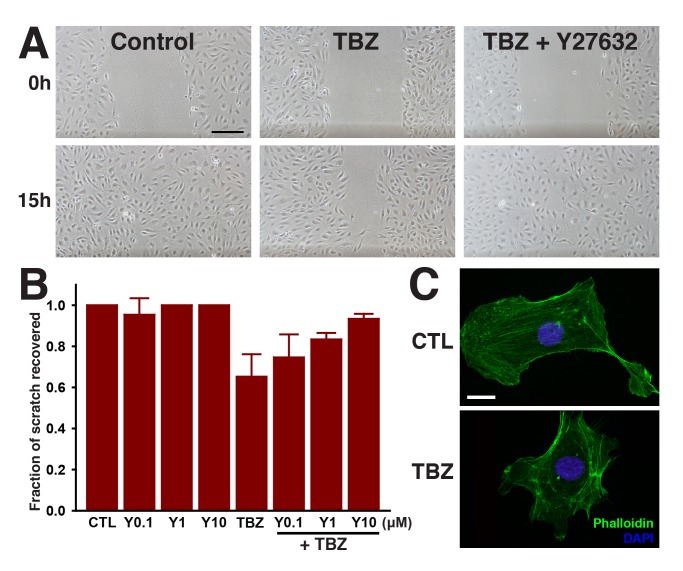
TBZ impedes migration of HUVECs in a wound scratch assay, but treatment with the Rho Kinase inhibitor Y27632 reverses TBZ's effects. (A) The effects of 1% DMSO-treated control versus 1% DMSO, 250 µM TBZ, and 1% DMSO, 250 µM TBZ, 10 µM Y27632. Scale bar, 200 µm. (B) quantifies the dose-dependent suppression of TBZ inhibition by Y27632. Error bars represent the mean ± 1 s.d. across 3 wells (1 of 3 trials). TBZ results in disorganization of actin stress fibers, as shown in (C) for 1% DMSO-treated control versus 1% DMSO, 250 µM TBZ-treated cells. Scale bar, 20 µm.

Finally, we found that treatment with TBZ significantly slowed endothelial cell migration in a scratch wound assay using cultured HUVECs (Figure 7AB). This quantitative in vitro assay with mammalian cells, combined with our in vivo data from *Xenopus*, demonstrate that TBZ disrupts established vasculature not by eliciting cell death but rather by perturbing endothelial cell behavior.

### Thiabendazole Acts via Tubulin and Rho Kinase to Control Endothelial Cell Behavior

The effect of TBZ on endothelial cells is striking and rapid. Our in vivo imaging of the vasculature revealed that endothelial cells retract from one another and round up within 2 h of TBZ treatment (Figures 5 and 6). Moreover, we observed that this effect is reversible by washout within a similarly rapid time frame (Figure 6). The rapid time-frames observed here argue that TBZ may act at the level of the cytoskeleton to influence endothelial cell behavior.

We first considered that, while not an assumption of the phenolog approach (see above), TBZ may nonetheless impact the vasculature by the same mechanism as lovastatin. Lovastatin disrupts angiogenesis at least in part by perturbing the geranyl-geranylation of the RhoA GTPase, thereby abrogating its activity [10]. RhoA is a critical regulator of actin-based behaviors in all animal cells [26], and the loss of RhoA signaling in endothelial cells treated with lovastatin is directly linked to cytoskeletal changes and inhibition of angiogenesis [10]. Indeed, inhibition of angiogenesis by lovastatin can be overcome by addition of geranyl-geranyl pyrophosphate (GGPP; [9],[10]). We therefore used the HUVEC scratch-wound closure model to quantitatively assess the effects of GGPP addition on TBZ action. However, we found that addition of GGPP did not reverse the action of TBZ on HUVEC cell motility in this assay (Figure S10). Similarly, while TBZ has been observed to affect the activity of porcine heart mitochondria [27], we detected no differences in mitochondrial mass (measured by MitoTracker Green signal) or mitochondrial membrane potential (measured as the ratio of MitoTracker Red signal to Mitotracker Green signal) (unpublished data), thus ruling out this potential activity as being relevant.

We next considered the possibility that TBZ acted on the vasculature at the level of the microtubule (MT) cytoskeleton, because TBZ has been found to disrupt microtubule assembly and dynamics in a number of cell types (e.g., [28]–[31]), and because several currently-studied VDAs act as MT-disrupting agents [21],[32]. Curiously, TBZ had only a very slight effect on the gross organization of the MT cytoskeleton in HUVEC cells in culture (Figure S11A), but a quantitative analysis using mass-spectrometry revealed a significant reduction in the abundance of several tubulin proteins following treatment of HUVECs with TBZ (Figure S11B).

Many MT-targeting VDAs act via hyper-activation of Rho signaling [25],[33],[34], likely reflecting the key role of MT-binding RhoGEFs [35]. We reasoned, therefore, that TBZ may also act via increased Rho signaling, as the drug elicited several phenotypes known to be associated with dysregulated Rho signaling (e.g., cell rounding, re-distribution of actin filaments, and defects in cell motility; Figure 7). To test this model directly, we asked if disruption of Rho signaling might counteract the effects of TBZ. Indeed, pharmacological disruption of Rho kinase function using the small molecule Y27632 elicited a significant and dose-dependent rescue of the TBZ-induced HUVEC cell motility defect (Figure 7A,B). Together, these data suggest that vascular disruption by TBZ results from reduced tubulin levels and hyper-active Rho signaling.

### Thiabendazole Slows Tumor Growth in a Mouse Xenograft Model

**Figure 8:**
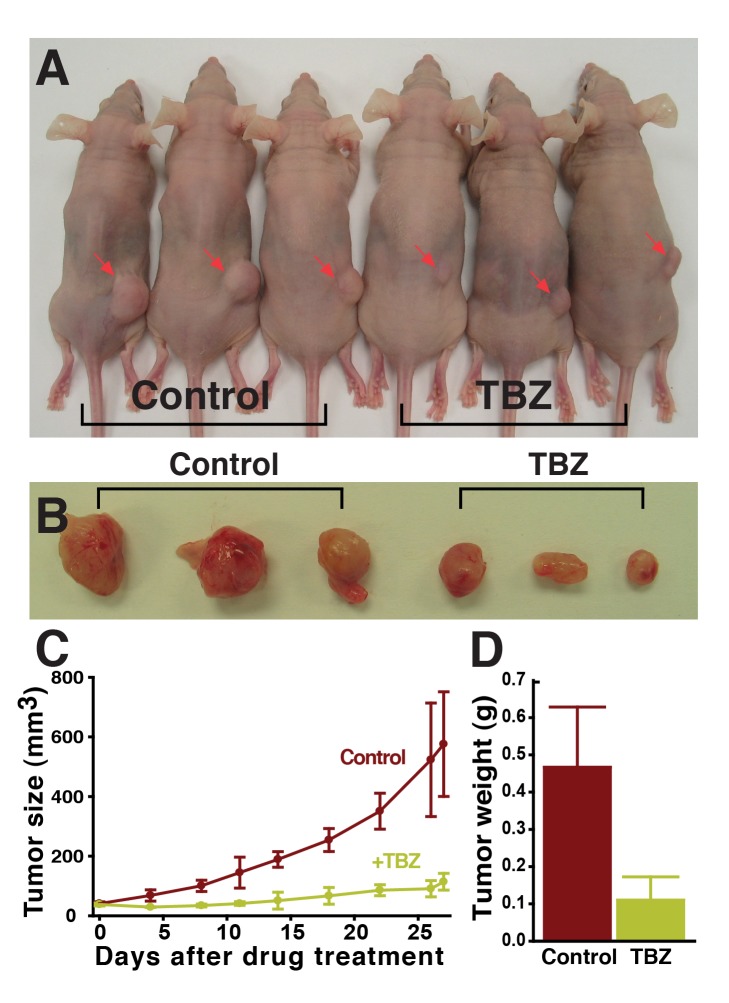
TBZ slows the growth of human HT1080 fibrosarcoma xenograft tumors in athymic Cre nu/nu mice. Tumors are significantly reduced in size in TBZ-treated animals (A), shown in (B) biopsied from mice after 27 d of 50 mg/kg (corresponding to 250 µM) TBZ treatment, and quantified in (C) and (D) (1 of 2 trials).

It is hoped that VDAs may open new therapeutic avenues by complementing the action of currently used angiogenesis inhibitors (e.g., [4]). Moreover, the data above suggest that the mechanism of TBZ action distinguishes it from VDAs such as ASA404, which act by inducing endothelial cell apoptosis [24], but which failed to show efficacy in a recent Phase III clinical trial for treatment of lung cancer [36]. To begin to ask if TBZ may be useful in the arena of cancer therapy, we tested the ability of TBZ to slow the growth of solid vascularized tumors in a mammal. We therefore employed a mouse xenograft model typical of those proven valuable in indicating the effectiveness of anti-angiogenesis therapy [37],[38]. We found that TBZ treatment significantly slowed HT1080 human fibrosarcoma xenograft growth in athymic Cre nu/nu mice [39], as assessed by a time course of tumor size and also by final tumor mass (Figure 8).

**Figure 9:**
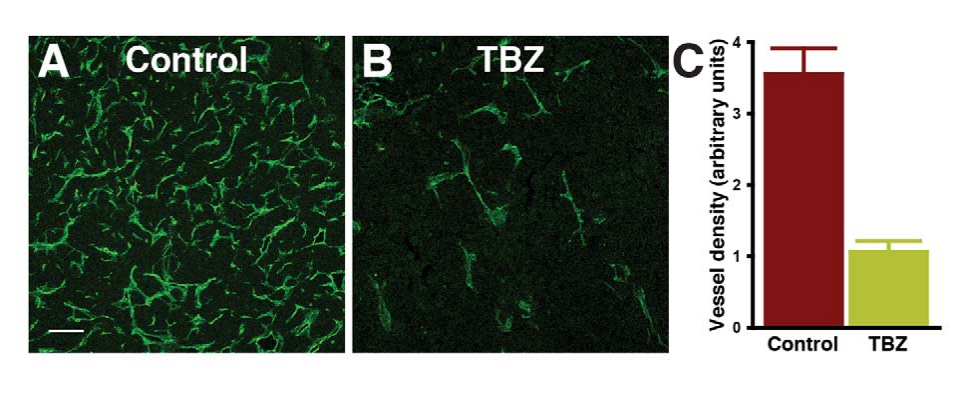
Blood vessel density is significantly reduced within TBZ-treated tumors. (A) and (B) show tumor vasculature visualized by immunohistochemistry of microdissected tumor sections using an anti-CD31 (PECAM-1) antibody staining for vasculature in the region of highest vessel density (“hot spots”; scale bar, 100 µm), and the total area of PECAM-1 staining above a fluorescence intensity threshold (arbitrary units) is quantified in (C).

Our in vivo data from *Xenopus*, as well as our human in vitro data, suggest that TBZ likely slows tumor growth by acting at the level of the vasculature (Figures 3 and 4). Consistent with this model, TBZ treatment did not alter the rate of proliferation in HT1080 cells when cultured in vitro but did significantly impair tumor microvessel density in xenografts (Figures 9 and S12). In addition, we noted that treatment with TBZ did not alter the levels of VEGF expressed or secreted by HT1080 cells, consistent with it acting downstream of VEGF in tumor xenografts (Figure S13), as it does in developing *Xenopus* embryos in vivo (Figure S3). Notably, we employed a TBZ dose of 50 mg/kg for these experiments, which is concordant with the FDA-approved maximum recommended daily dose of TBZ in humans, suggesting the possibility of chemotherapeutic use in humans.

### Conclusions

In sum, an analysis of evolutionary repurposing of a genetic module shared from yeast to humans has led directly to the discovery that an orally available drug, thiabendazole, already FDA approved for clinical use in humans, also acts as an angiogenesis inhibitor and vascular disrupting agent. Moreover, these data establish TBZ as the only VDA currently approved for human use (albeit for a different purpose). Our data suggest that, even for antifungal or antihelmintic use, the possibility of side effects related to vascularization should be considered, for example, in patients with cardiovascular disease or to the fetus if administered to pregnant women, for whom TBZ has not been broadly tested. Significantly, while research on VDAs has largely centered on cancer therapy, their use may also provide new therapeutic avenues for non-malignant diseases, such as diabetic retinopathy and macular degeneration [40],[41]. With more than 40 years of human use, the low cost and generic availability of TBZ make it a compelling candidate for translation into the clinic as a VDA.

Finally, this research emphasizes the advantages of an evolutionary approach to drug discovery, in which the natural experimental strengths of various organisms can be exploited to accelerate our understanding of a conserved genetic module. Importantly, this approach proceeded from a gene module-based discovery strategy and proved effective even though the associated organismal phenotypes were entirely unrelated. Curiously, at least two other known antifungal drugs can also act as angiogenesis inhibitors. Itraconazole, an azole antifungal drug otherwise structurally unrelated to TBZ and acting via different mechanisms, was identified as an angiogenesis inhibitor via high-throughput screening [42]. This observation, in addition to the dual anti-fungal and anti-angiogenic properties of lovastatin [9],[10],[43], suggests additional interesting evolutionary connections between the processes of yeast cell wall metabolism and vertebrate angiogenesis. Such evolutionary connections further support the yeast cell wall-relevant activity of TBZ, from among its multiple pharmacological targets, as being the relevant activity for angiogenesis. Thus there is no reason to suspect that a more highly targeted agent could not be repurposed in a similar fashion. Overall, these results suggest that a fundamental understanding of systems biology will prove to be directly relevant to drug discovery, complementing traditional screening approaches to pharmacophore discovery and accelerating both basic and clinical biomedical research.

## Materials and Methods

### Clustering Analysis

Compound genetic interaction profiles were downloaded from http://chemogenomics.stanford.edu:16080/supplements/global/download.html. We employed the *p* values reported for fitness defects in the yeast homozygous deletion collection for all analyses [13]. Candidate angiogenesis inhibitors were prioritized that consistently clustered with lovastatin across different choices of similarity measures and hierarchical clustering algorithms, specifically centered and uncentered correlation, Spearman rank correlation, absolute correlation (centered and uncentered), Euclidean distance, and City-block distance, employing centroid linkage, complete linkage, single linkage, or average linkage clustering. Clustering results were visualized with Cluster 3.0 (http://bonsai.hgc.jp/~mdehoon/software/cluster/software.htm) and Java TreeView (http://jtreeview.sourceforge.net/).

### *Xenopus* Embryo Manipulations

Female adult *Xenopus* were ovulated by injections of human chorionic gonadotropin, and eggs were fertilized in vitro and dejellied in 3% cysteine (pH 7.9) and subsequently reared in 1/3× Marc's modified Ringer's (MMR) solution. For microinjections, embryos were placed in a solution of 2% Ficoll in 1/3× MMR solution, injected using forceps and an Oxford universal manipulator, reared in 2% Ficoll in 1/3× MMR to stage 9, then washed and reared in 1/3× MMR solution alone. For bilateral *rab11b* knock-down experiments, the posterior cardinal vein and intersomitic veins were targeted by injecting Morpholino antisense oligonucleotides (MOs) into the two ventral cells equatorially at the four-cell stage. For unilateral knockdown, only one ventral cell was injected. MOs were injected at 40 ng per blastomere. For the experiments to see the drug effects, embryos were placed in a solution of each chemical dissolved in 1% DMSO diluted in 1/3× MMR during indicated stages. For bead micro-surgery implantation, Affi-Gel Blue Gel beads (Bio-Rad) were soaked with 0.7 mg/ml recombinant mouse *VEGF* 164 aa (R&D systems) or BSA as a control. Whole-mount in situ hybridization for *erg* and *aplnr* was performed as described [44].

### Morpholino Oligonucleotides and cDNA Clones

*Erg* and *aplnr* cDNAs were obtained from Open BioSystems (*erg*: IMAGE:5512670, *aplnr*: IMAGE:8321886). Translation-blocking antisense morpholinos for *rab11b* were designed based on the sequences from the National Center for Biotechnology Information database (accession number: BC082421.1). MOs were obtained from Gene Tools with the following sequence: 5′-CGTATTCGTCATCTCTGGCTCCCAT-3′.

### Cell Culture

Human umbilical vein endothelial cells (HUVECs) were purchased from Clonetics, and were used between passages 4 and 9. HUVECs were cultured on 0.1% gelatin-coated (Sigma) plates in endothelial growth medium-2 (EGM-2; Clonetics) in tissue culture flasks at 37°C in a humidified atmosphere of 5% CO_2_.

### In Vitro Angiogenesis Assays

HUVECs (10^4^ cells) were seeded in a 96-well plate coated with 50 µl of ECMatrix (Chemicon) or Matrigel (BD Bioscience) according to the manufacturer's instructions. Cells were incubated for 16 h on EGM-2 containing thiabendazole, dissolved in 1% DMSO. Negative control cells were treated with 1% DMSO in the same manner. As a positive control, siRNA versus the human HoxA9 sequence [45] was transfected into HUVECs using Lipofectamine RNAiMAX (Invitrogen) according to the manufacturer's instructions. Tube formation was observed using an inverted microscope (Nikon, eclipse TS100), and branch points were measured using ImageJ software (http://rsb.info.nih.gov/ij).

### Cell Migration Assays

HUVECs (1.2×10^5^ cells) were seeded into 24-well plates for 24 h, and the monolayers were wounded identically. Then, cells were washed with PBS and treated with EBM-2 containing 1% DMSO or 250 µM TBZ dissolved in 1% DMSO with a combination of Y27632 or GGPP (Sigma). In the case of Y27632 treatment, cells were preincubated for 2 h before wounding. Cells were photographed at time zero and after 15 h, and the ratios of cell free area [(0 h–15 h)/0 h] were calculated.

### Xenograft Model

Specific pathogen-free athymic Cre nu/nu mice were purchased from Charles River Laboratories. The HT1080 human fibrosarcoma cell line was obtained from the American Type Culture Collection (ATCC). HT1080 cells were cultured in DMEM (Gibco) containing 10% fetal bovine serum (FBS, Gibco) in tissue culture flasks at 37°C in a humidified atmosphere of 5% CO_2_. In order to generate a mouse xenograft model, a suspension of the HT1080 cells (3×10^6^ in 50 µl PBS) mixed with an equal volume of Matrigel (BD Bioscience) was subcutaneously implanted into the flank region of 7–8-wk-old female mice. Upon establishment of tumors (approx. 40 mm^3^), mice were given daily intraperitoneal injections of 1 mg thiabendazole (Sigma-Aldrich), suspended in 20 µl DMSO. Mice weighed on average 20 grams; this dose thus corresponded to 250 µM TBZ. As a control, an equal volume of DMSO was injected in the same manner. Tumor growth was monitored by measuring the length and width of each tumor using digital calipers, and the tumor volume in mm^3^ calculated by the formula: Volume = (width)^2^×length/2. Upon a tumor reaching the maximum size permitted by the Institutional Animal Care and Use Committee (1.5 cm in diameter), the mouse was sacrificed, and the tumor excised.

### Immunohistochemistry

Each tumor was fixed with 4% paraformaldehyde in PBS, and cryostat sections were processed. After blocking with 5% goat serum in PBST (0.3% Triton X-100 in PBS) for 1 h at room temperature, sectioned tissues were incubated with anti-mouse CD31 antibody, hamster clone 2H8, 1∶100 (Millipore). After several PBST washes, samples were incubated for 2 h at room temperature with FITC-conjugated anti-hamster IgG antibody, 1∶1,000 (Jackson ImmunoResearch).

In order to determine the effect of thiabendazole on proliferation and apoptosis, 2×10^5^ HUVECs or HT1080 were cultured in 6-well plates and treated with thiabendazole dissolved in 1% DMSO. Control cells received 1% DMSO. For actin and tubulin cytoskeleton analysis, 7×10^4^ HUVECs were seeded. After 24 h, cells were fixed using 4% paraformaldehyde in PBS. Cell membranes were permeabilized with 0.2% Triton X-100 in PBS, and nonspecific immunobinding sites were blocked with 5% goat serum for 1 h at room temperature. Cells were incubated with primary antibodies to Caspase-3 (Abcam), Phospho-histone H3 (Ser10; Millipore), or β-tubulin (Sigma) at 4°C overnight. After washing with PBST, primary antibodies were detected by Alexa Fluor-488 or 555 goat anti-rabbit immunoglobulin (IgG). Alexa Fluor 488 phalloidin (Invitrogen) and/or 4′,6-Diamidino-2-phenylindole (Sigma) were added as needed.

Immunostaining for *Xenopus* was performed as previously described [46]. Embryos at stage 35–36 were fixed in 1× MEMFA. 12/101 (1∶500; DSHB) and primary antibodies were detected with Alexa Fluor-488 or 555 goat anti-mouse Immunoglobulin (IgG).

### Imaging and Image Analysis

Immunohistochemistry experiments and *kdr:GFP* transgenic *Xenopus laevis* were imaged on an inverted Zeiss LSM5 Pascal confocal microscope and Zeiss 5-LIVE Fast Scanning confocal microscope. Confocal images were processed and cropped in Imaris software (BITPLANE) and Adobe Illustrator and Adobe Photoshop for compilation of figures.

### Mass Spectrometry

HUVECs were treated with 1% DMSO or 1% DMSO, 250 µM TBZ for 24 h, and lysed by Dounce homogenization in low salt buffer (10 mM Tris-HCl, pH 8.8, 10 mM KCl, 1.5 mM MgCl_2_) with 0.5 mM DTT and protease inhibitor mixture (Calbiochem). 2,2,2-trifluoroethanol was added to 50% (v/v) for each sample, and samples were reduced with 15 mM DTT at 55°C for 45 min and then alkylated with 55 mM iodoacetamide at room temperature for 30 min. Following alkylation, samples were diluted in digestion buffer (50 mM Tris-HCl, pH 8.0, 2 mM CaCl_2_) to a final 2,2,2-trifluoroethanol concentration of 5% (v/v) and digested using proteomics grade trypsin (Sigma) at 1∶50 (enzyme/protein) concentration and incubated at 37°C for 4–5 h. Digestion was halted with the addition of 1% formic acid (v/v), and sample volume was reduced to 200 µl by SpeedVac centrifugation prior to loading on HyperSep C-18 SpinTips (Thermo). Samples were eluted (60% acetonitrile, 0.1% formic acid), reduced to 10 µl by SpeedVac centrifugation, and resuspended in sample buffer (5% acetonitrile, 0.1% formic acid). Tryptic peptides were then filtered through Microcon 10-kDa centrifugal filters (Millipore), and collected as flow-through. Peptides were chromatographically separated on a Zorbax reverse-phase C-18 column (Agilent) via a 230 min 5%–38% acetonitrile gradient, then analyzed by on-line nanoelectrospray-ionization tandem mass spectrometry on an LTQ-Orbitrap (Thermo Scientific). Data-dependent ion selection was performed, collecting parent ion (MS1) scans at high resolution (60,000) and selecting ions with charge >+1 for collision-induced dissociation fragmentation spectrum acquisition (MS2) in the LTQ, with a maximum of 12 MS2 scans per MS1. Ions selected more than twice in a 30 s window were dynamically excluded for 45 s. MS2 spectra were interpreted using SEQUEST (Proteome Discoverer 1.3, Thermo Scientific), searching against human protein-coding sequences from Ensembl release 64 [47]. Search results were then processed by Percolator [48] at a 1% false discovery rate. Protein groups were generated comprising proteins with identical peptide evidence, omitting those proteins whose observed peptides could be entirely accounted for by other proteins with additional unique observations. Differential expression of proteins across TBZ-treated and control samples was quantified from the MS2 spectral count data using the APEX method of relative quantification [49].

### Western Blotting and ELISA

HT1080 (2×10^5^ or 4×10^4^cells) were cultured in 6-well plates and treated with 1% DMSO or 1% DMSO, 250 µM TBZ for 24 h. Cells were lysed in cell lysis buffer (Cell Signaling Technology) containing 1 mM PMSF, and analyzed by SDS-PAGE and Western blotting using anti-VEGF (Santa Cruz, A-20) or anti-GAPDH (Cell Signaling Technology) antibodies. The secreted VEGF level in culture medium was determined by enzyme-linked immunosorbent assay (ELISA; R&D) according to the manufacturer's instructions.

## Supporting Information

Figure S1 Unilateral morpholino (MO) knockdown of *rab11b* induces vascular defects in developing *Xenopus laevis* (frog) embryos, showing the control versus knockdown sides of the same animal and measured by in situ hybridization versus marker gene *aplnr*. ISV, intersomitic vein; PCV, posterior cardinal vein.
(TIF)

Figure S2 In yeast chemical genetic interaction datasets [13], TBZ treatment consistently clustered with lovastatin treatment across different choices of similarity measures and clustering algorithms. Three cases out of 19 trials are illustrated here, organized as in Figure 2C.
(TIF)

Figure S3 TBZ inhibits ectopic angiogenesis (note the doubled PCV in the left panel) stimulated by Affy-gel blue beads (75–150 µm diameter, indicated by white arrowheads) pre-soaked with 0.7 mg/ml vascular endothelial growth factor (VEGF) and microsurgically implanted into developing *Xenopus* embryos, assaying for the vasculature by ISH versus *erg* or *aplnr* (showing data for *erg*). 6 of 8 control animals developed ectopic PCV or VV, as opposed to 0 of 9 TBZ-dosed animals (*p* value = 0.0023). TBZ-treated embryos show notably disorganized (cellularized) vasculatures.
(TIF)

Figure S4 Somitic muscle, defined with the 12/101 antibody, on 1% DMSO, 250 µM, TBZ-treated *Xenopus* embryo, is normal compared to 1% DMSO control. Both were treated at stage 31 and imaged at stages 35–36.
(TIF)

Figure S5 Tests of commercially available TBZ variants indicate that in vivo angiogenesis inhibition activity in *Xenopus* varies strongly across benzimidazoles and suggests necessary chemical moieties (for example, suggesting that the thiazole group, or at least its nitrogen, is important to activity).
(TIF)

Figure S6 TBZ treatment shortly after the posterior cardinal vein (PCV) is established (stage 36) causes *Xenopus* vascular structures to re-cellularize in vivo, shown by in situ hybridization versus *aplnr* at stage 39.
(TIF)

Figure S7 Blood vessel development visualized in vivo using time-lapse fluorescence microscopy of the vasculature developing within a living *Xenopus* embryo. Arteries and veins are visualized as in Figures 3 and 5 by vascular-specific *kdr:GFP* frogs from [19], showing the vasculature of stage 46 animals treated from stage 41 with the 1% DMSO control. Scale bar, 80 µm.
(TIF)

Figure S8 Retraction and rounding of vascular endothelial cells (blue arrowheads) is apparent at higher magnification in TBZ-treated embryos (as in Figures 3 and 5). Scale bar, 30 µm.
(TIF)

Figure S9 TBZ treatment (A) increases apoptosis and (B) decreases proliferation of HUVEC cells cultured on 0.1% gelatin, but only by approximately 2-fold. Both changes are significant under a *t* test (*p* = 0.015, *p* = 0.01, respectively). Error bars represent mean ± 1 s.d. across 14–16 fields of view of 200× magnification confocal microscopy cell images across 2–3 independent experiments. Scale bar, 100 µm.
(TIF)

Figure S10 (A, B) GGPP does not reverse the impeded migration of HUVECs in a wound-scratch assay. (A) shows effects of 1% DMSO-treated control versus 1% DMSO, 250 µM TBZ, and 1% DMSO, 250 µM TBZ, 25 µM GGPP. (B) shows quantification as a function of varying GGPP concentrations. Error bars represent mean ± 1 s.d. across 3 wells (1 of 2 trials). Scale bar, 200 µm.
(TIF)

Figure S11 Immunohistochemical analysis of β-tubulin does not show a definite distinction between 1% DMSO-treated control and 1% DMSO, 250 µM TBZ-treated HUVECs (A), but tubulins in HUVECs identified by a quantitative mass-spectroscopy analysis were significantly reduced with TBZ treatment (B). Scale bar in (A), 20 µm.
(TIF)

Figure S12 TBZ treatment does not significantly affect the proliferation of HT1080 cells. Error bars represent mean ± 1 s.d. across 14–15 fields of view of 200× magnification confocal microscopy cell images across 2 independent experiments. Scale bar, 100 µm.
(TIF)

Figure S13 TBZ does not significantly alter intracellular or secreted VEGF levels in HT1080 cells. VEGF levels in HT1080 cells and conditioned medium were measured in 1% DMSO-treated control and 1% DMSO, 250 µM TBZ-treated HT1080 cells, assaying cellular VEGF by Western blotting (A) and secreted VEGF by ELISA (B). Glyceraldehyde-3-phosphate dehydrogenase (GAPDH) level was measured for a Western blotting control.
(TIF)

Movie S1 In vivo vascular development in kdr:GFP frogs.
(M4V)

Movie S2 Disassembly of vasculature in *kdr:GFP* frogs following addition of 250 µM TBZ.
(M4V)

Movie S3 Disassembly of vasculature and reassembly after addition and then washout of 250 µM TBZ in *kdr:GFP* frogs.
(M4V)

Table S1 Conserved genes in the vertebrate angiogenesis defect/yeast lovastatin sensitivity gene module. Bold text indicates vertebrate genes whose angiogenesis roles were known or confirmed by the literature; italic text indicates genes whose roles were predicted in [1] and confirmed in frogs and HUVEC cells in [1] and Figures 2B and S1.
(DOC)

Table S2 Compounds computationally prioritized as candidate angiogenesis effectors. Nineteen alternate hierarchical clustering trials were performed varying the choice of clustering algorithm and the measure of similarity between drug-gene interaction profiles (from [13]), as described in Materials and Methods, and compounds were selected by the frequency with which they occurred in the same subcluster as lovastatin.
(DOC)

## Abbreviations

GFP: green fluorescent protein
GGPP: geranyl-geranyl pyrophosphate
HUVEC: human umbilical vein endothelial cell
MT: microtubule
TBZ: thiabendazole
VDA: vascular disrupting agent
