# Supplementary figures and images for: Evolutionarily Repurposed Networks Reveal the Well-Known Antifungal Drug Thiabendazole to Be a Novel Vascular Disrupting Agent
Source: PLoS Biol. 2012 Aug 21;10(8):e1001379. doi: 10.1371/journal.pbio.1001379 (PMC3423972; doi:10.1371/journal.pbio.1001379)

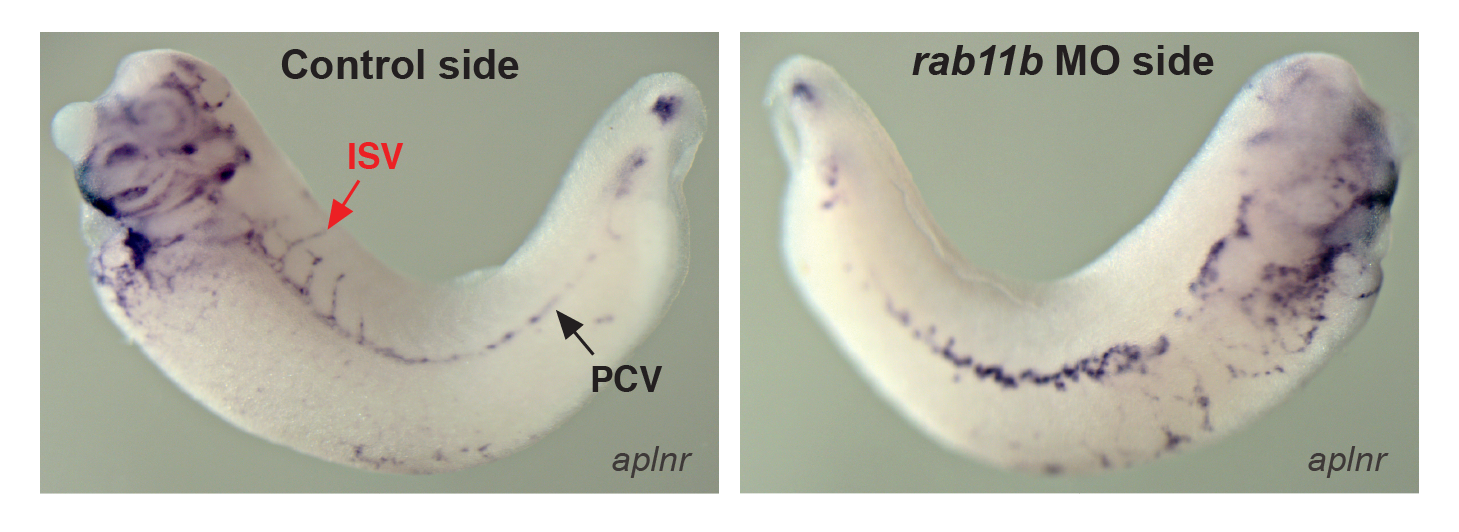

Supplement: Figure S1 — Unilateral morpholino (MO) knockdown of rab11b induces vascular defects in developing Xenopus laevis (frog) embryos, showing the control versus knockdown sides of the same animal and measured by in situ hybridization versus marker gene aplnr. ISV, intersomitic vein; PCV, posterior cardinal vein. (TIF) [file pbio.1001379.s001.tif]

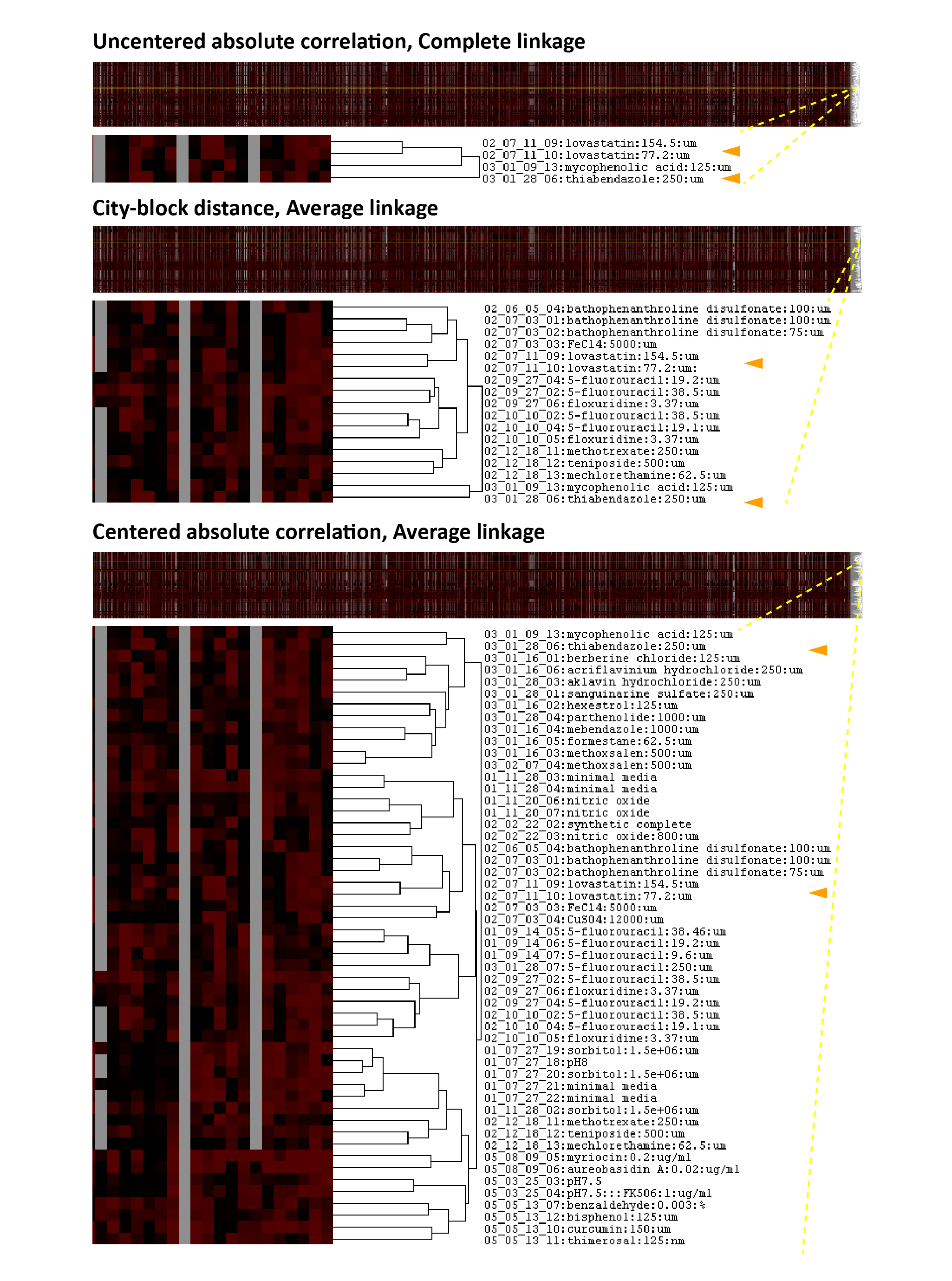

Supplement: Figure S2 — In yeast chemical genetic interaction datasets [13], TBZ treatment consistently clustered with lovastatin treatment across different choices of similarity measures and clustering algorithms. Three cases out of 19 trials are illustrated here, organized as in Figure 2C. (TIF) [file pbio.1001379.s002.tif]

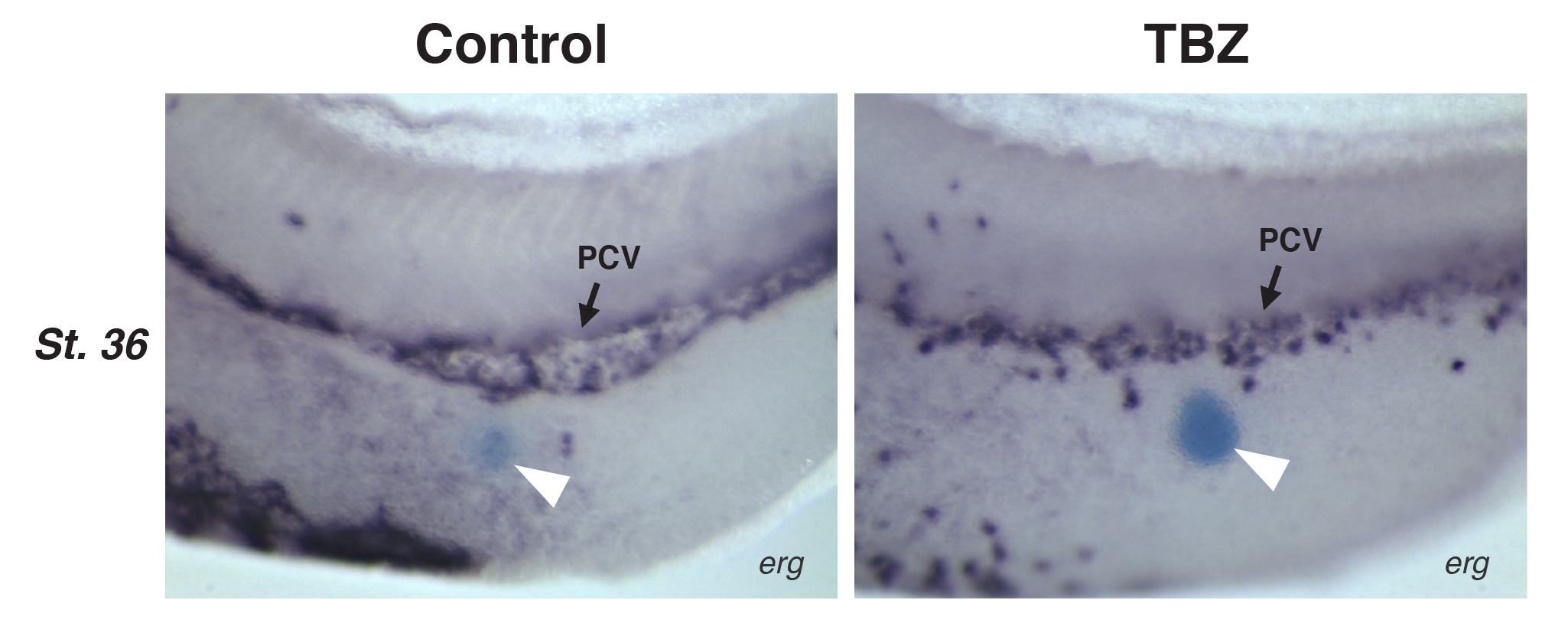

Supplement: Figure S3 — TBZ inhibits ectopic angiogenesis (note the doubled PCV in the left panel) stimulated by Affy-gel blue beads (75–150 µm diameter, indicated by white arrowheads) pre-soaked with 0.7 mg/ml vascular endothelial growth factor (VEGF) and microsurgically implanted into developing Xenopus embryos, assaying for the vasculature by ISH versus erg or aplnr (showing data for erg). 6 of 8 control animals developed ectopic PCV or VV, as opposed to 0 of 9 TBZ-dosed animals (p value = 0.0023). TBZ-treated embryos show notably disorganized (cellularized) vasculatures. (TIF) [file pbio.1001379.s003.tif]

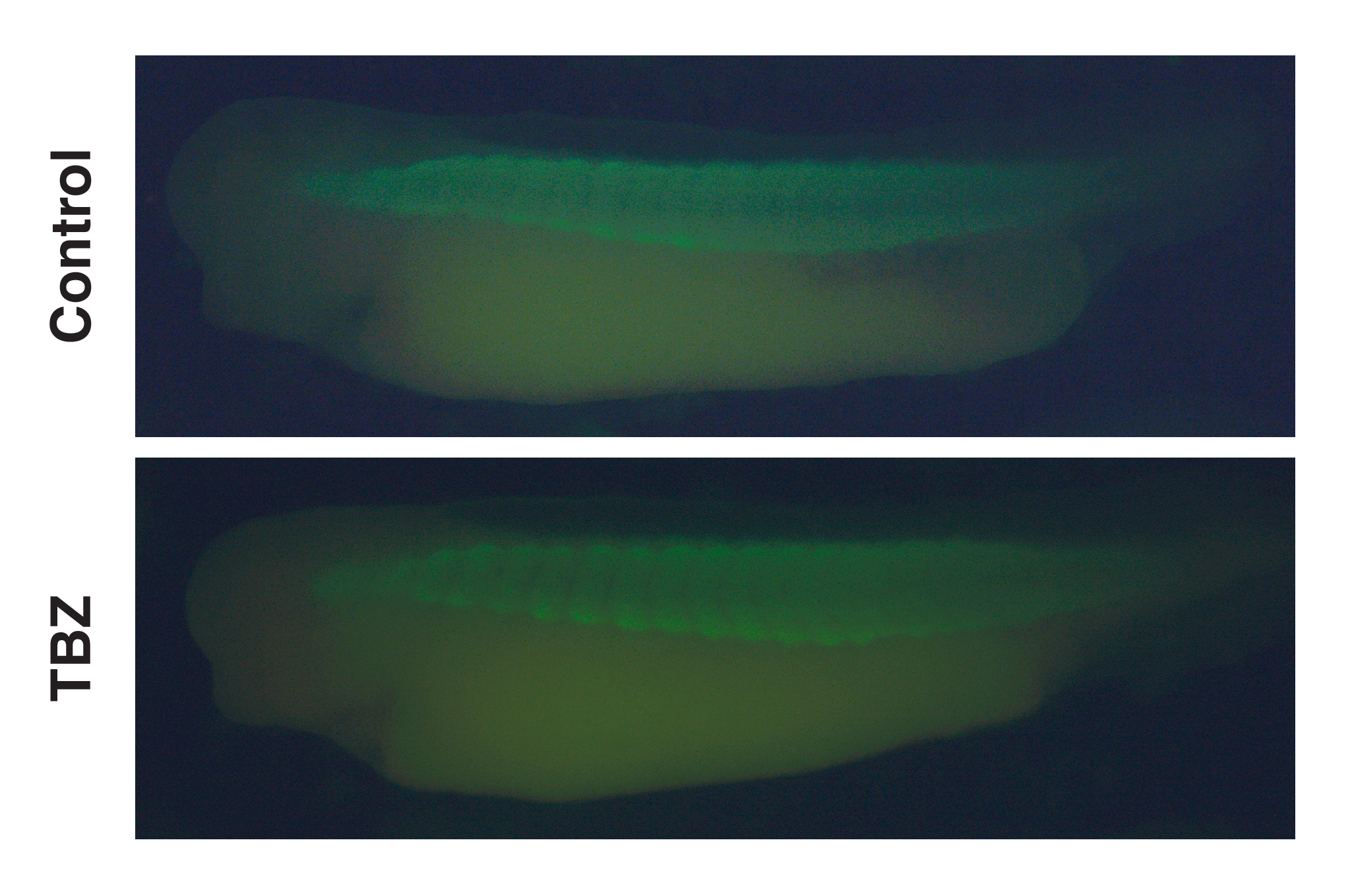

Supplement: Figure S4 — Somitic muscle, defined with the 12/101 antibody, on 1% DMSO, 250 µM, TBZ-treated Xenopus embryo, is normal compared to 1% DMSO control. Both were treated at stage 31 and imaged at stages 35–36. (TIF) [file pbio.1001379.s004.tif]

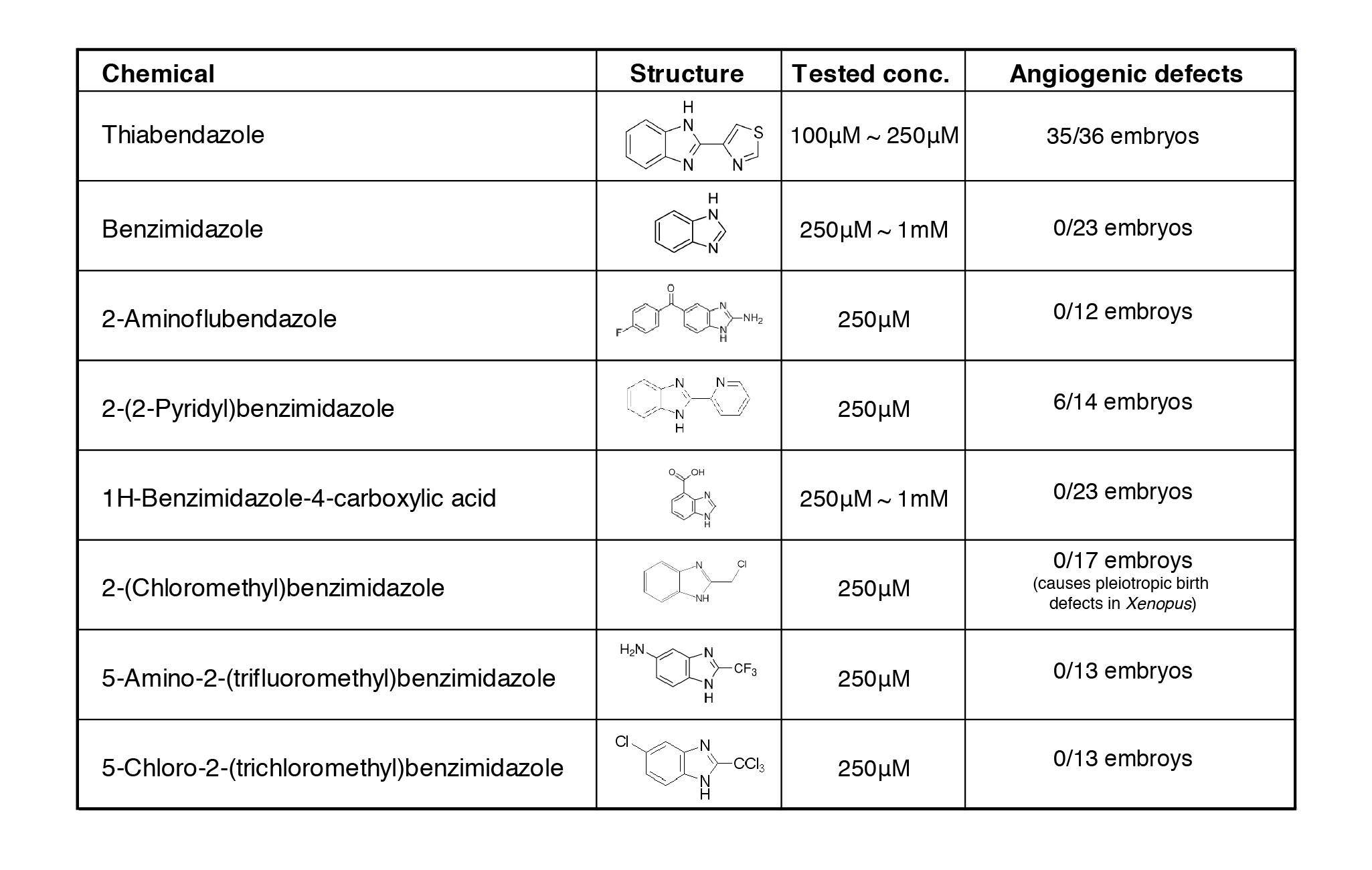

Supplement: Figure S5 — Tests of commercially available TBZ variants indicate that in vivo angiogenesis inhibition activity in Xenopus varies strongly across benzimidazoles and suggests necessary chemical moieties (for example, suggesting that the thiazole group, or at least its nitrogen, is important to activity). (TIF) [file pbio.1001379.s005.tif]

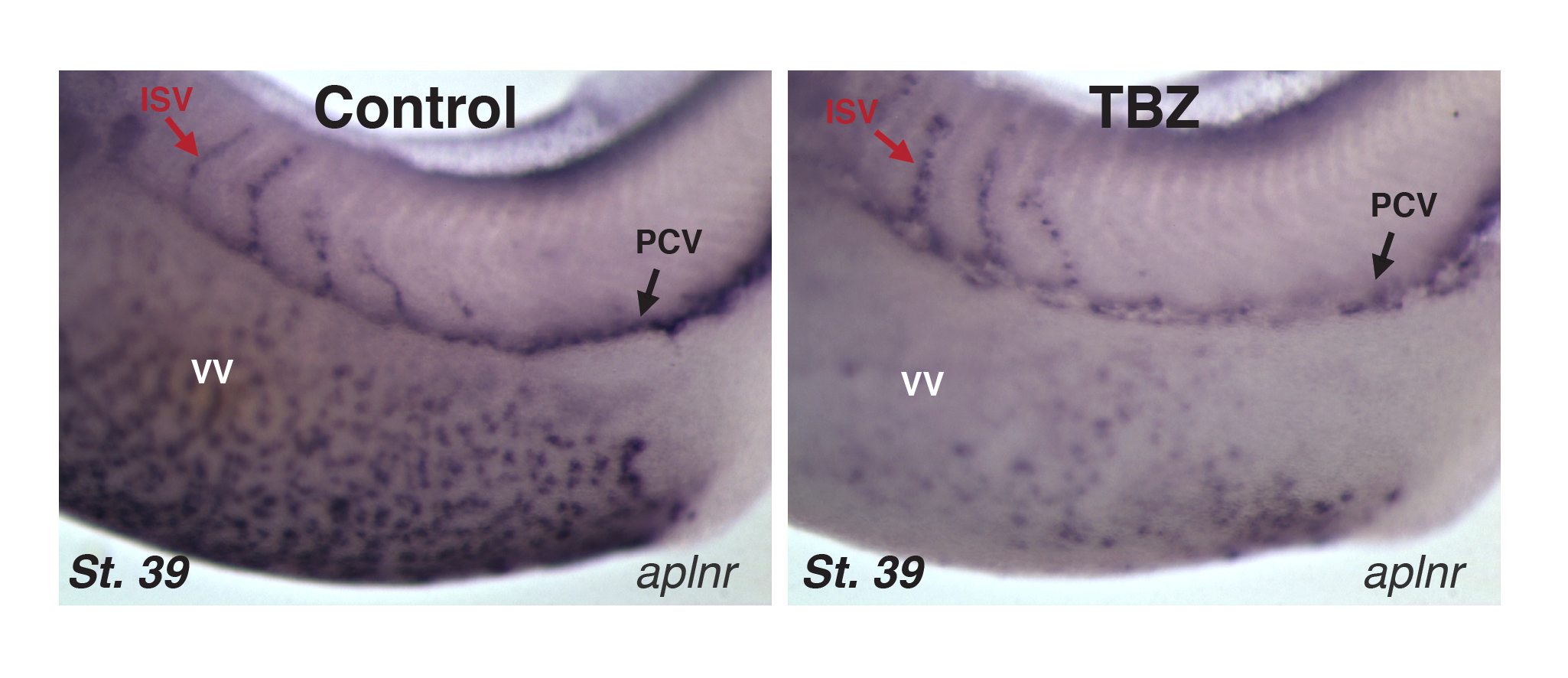

Supplement: Figure S6 — TBZ treatment shortly after the posterior cardinal vein (PCV) is established (stage 36) causes Xenopus vascular structures to re-cellularize in vivo, shown by in situ hybridization versus aplnr at stage 39. (TIF) [file pbio.1001379.s006.tif]

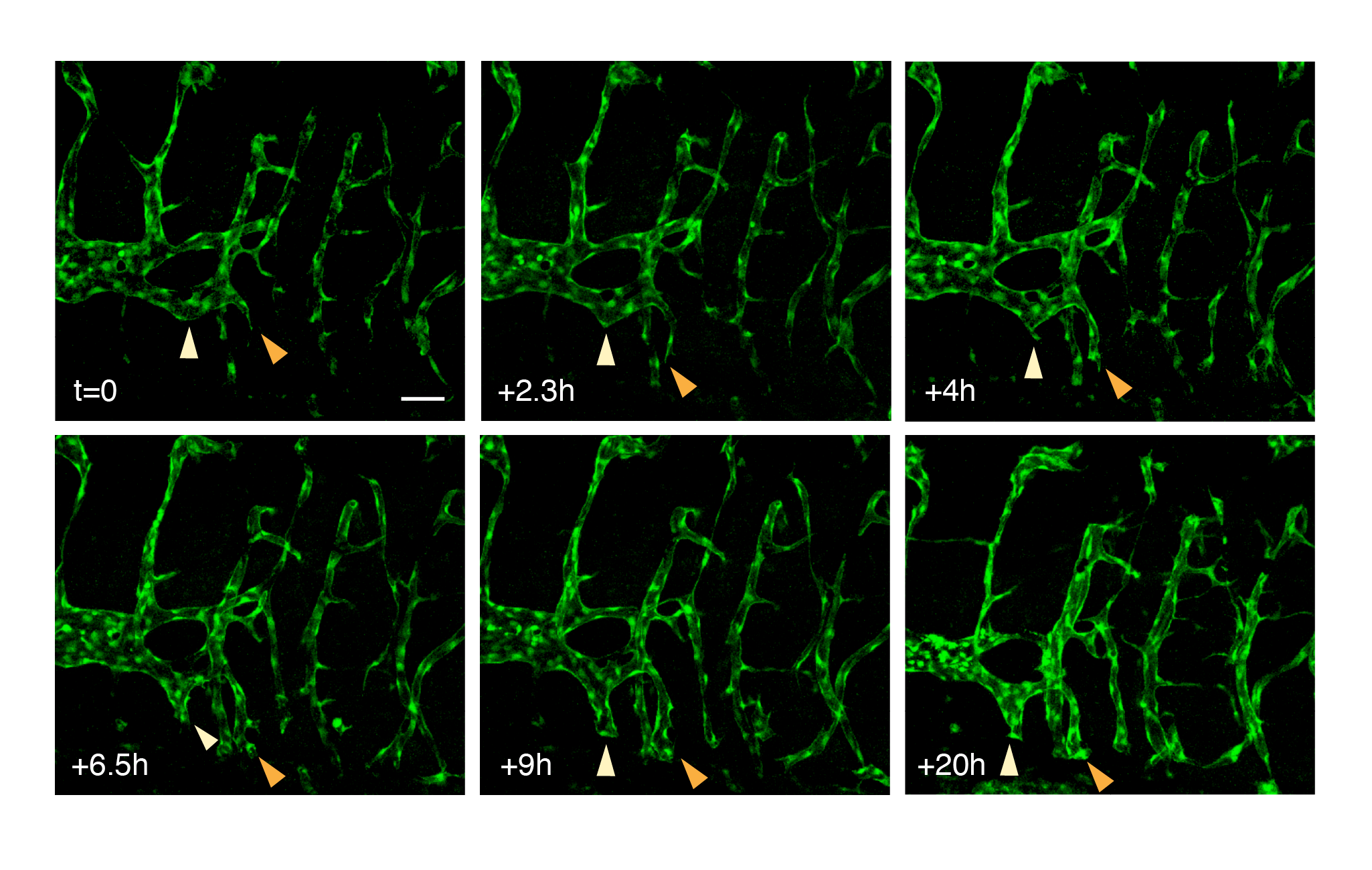

Supplement: Figure S7 — Blood vessel development visualized in vivo using time-lapse fluorescence microscopy of the vasculature developing within a living Xenopus embryo. Arteries and veins are visualized as in Figures 3 and 5 by vascular-specific kdr:GFP frogs from [19], showing the vasculature of stage 46 animals treated from stage 41 with the 1% DMSO control. Scale bar, 80 µm. (TIF) [file pbio.1001379.s007.tif]

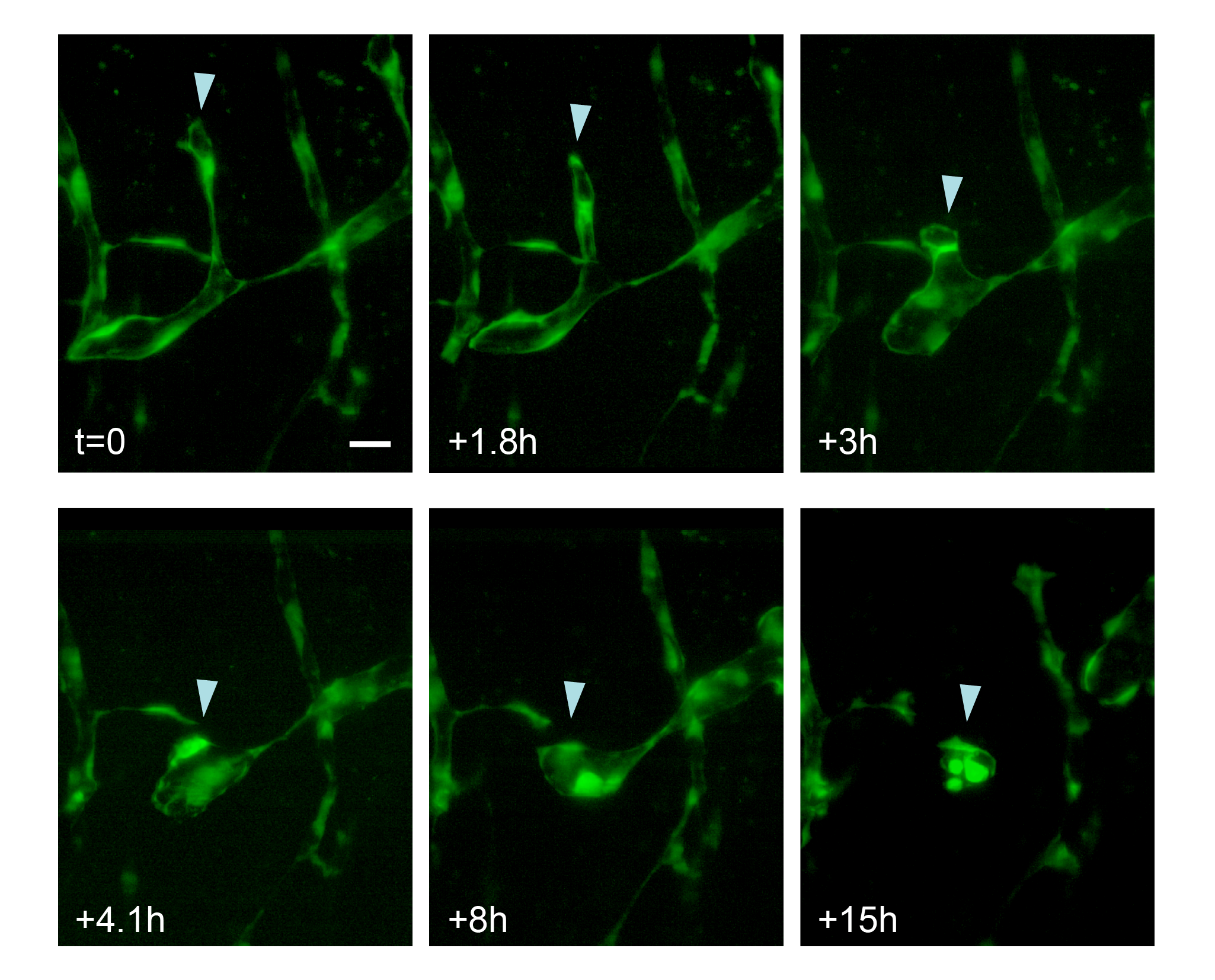

Supplement: Figure S8 — Retraction and rounding of vascular endothelial cells (blue arrowheads) is apparent at higher magnification in TBZ-treated embryos (as in Figures 3 and 5). Scale bar, 30 µm. (TIF) [file pbio.1001379.s008.tif]

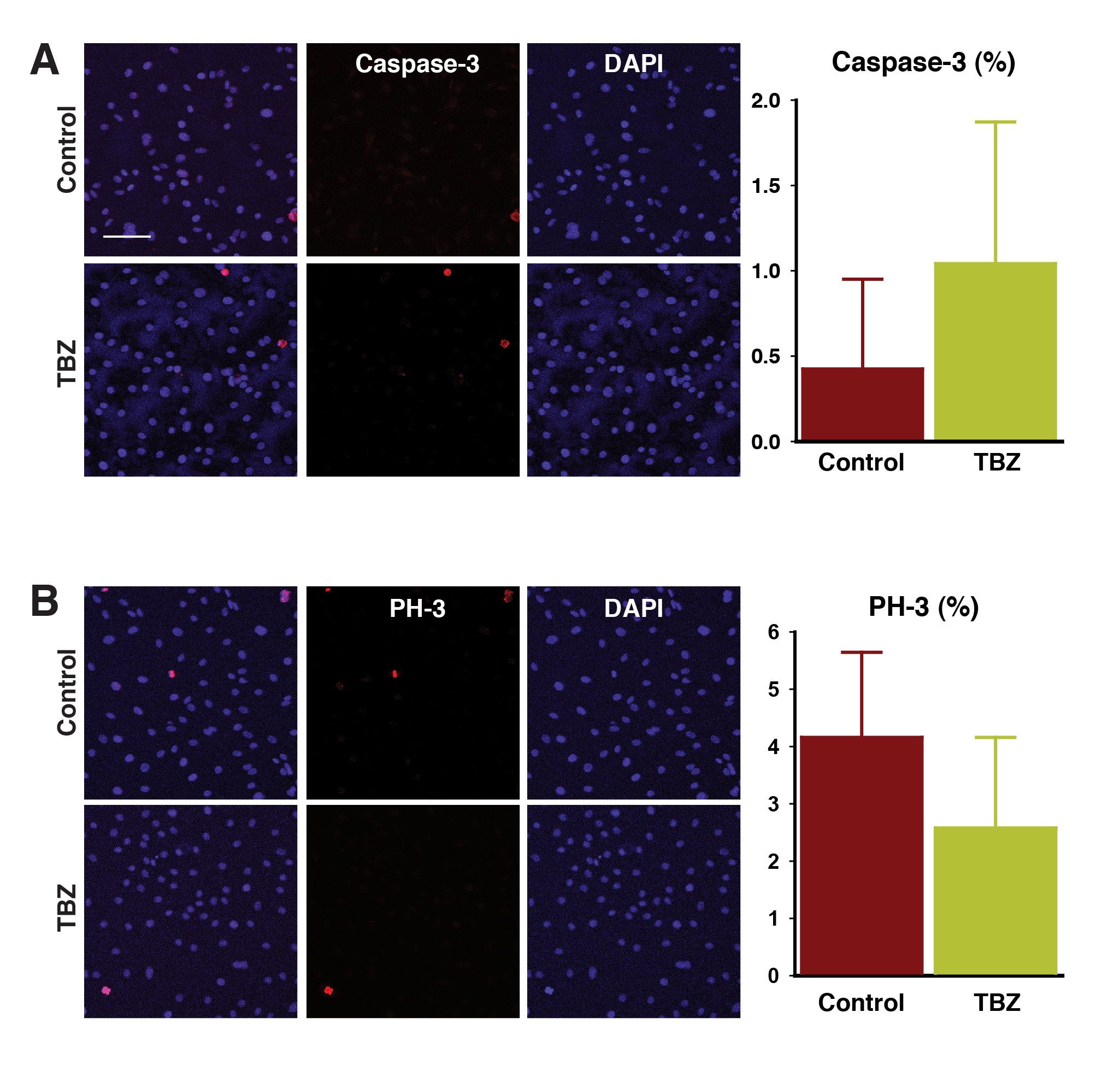

Supplement: Figure S9 — TBZ treatment (A) increases apoptosis and (B) decreases proliferation of HUVEC cells cultured on 0.1% gelatin, but only by approximately 2-fold. Both changes are significant under a t test (p = 0.015, p = 0.01, respectively). Error bars represent mean ± 1 s.d. across 14–16 fields of view of 200× magnification confocal microscopy cell images across 2–3 independent experiments. Scale bar, 100 µm. (TIF) [file pbio.1001379.s009.tif]

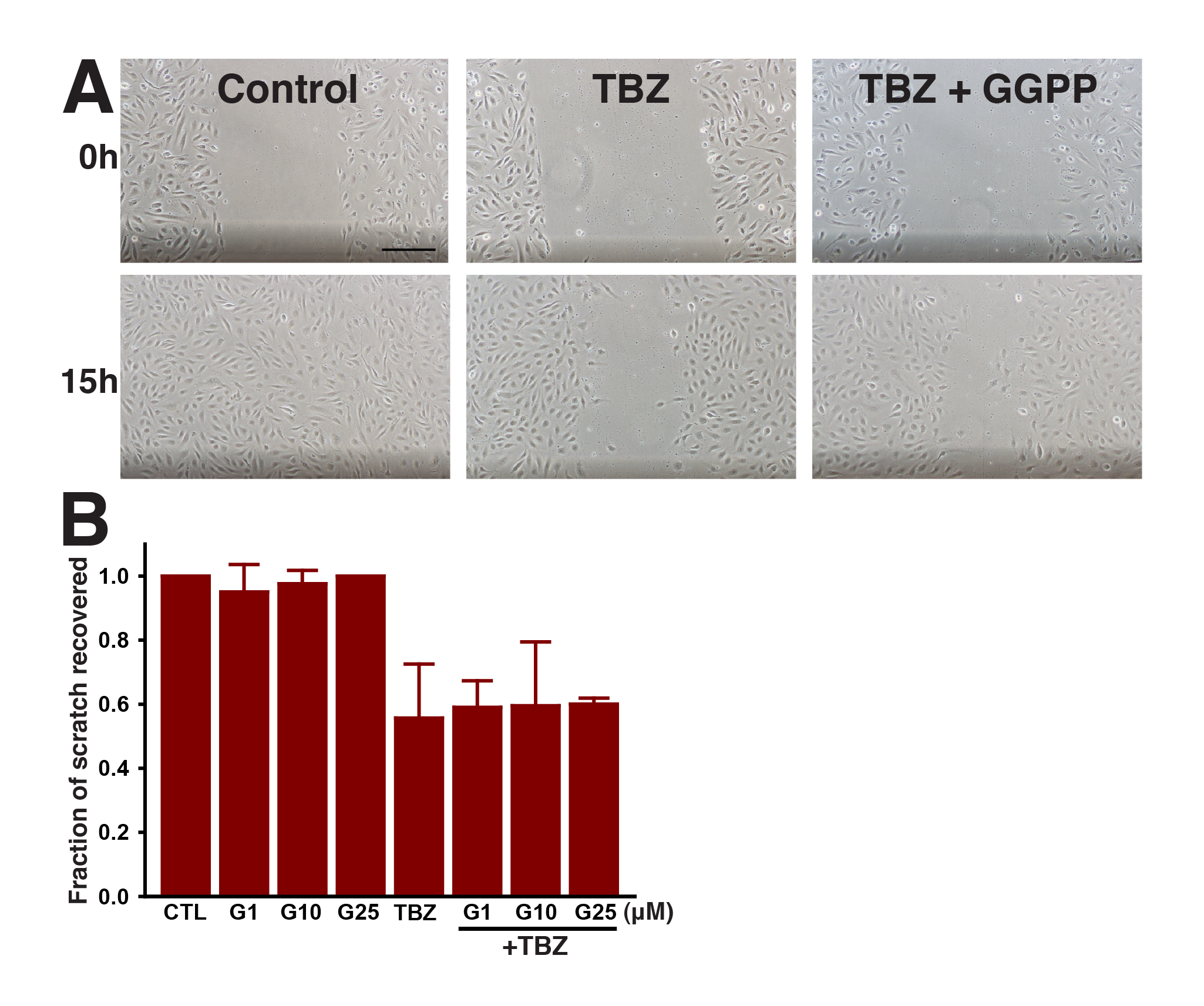

Supplement: Figure S10 — (A, B) GGPP does not reverse the impeded migration of HUVECs in a wound-scratch assay. (A) shows effects of 1% DMSO-treated control versus 1% DMSO, 250 µM TBZ, and 1% DMSO, 250 µM TBZ, 25 µM GGPP. (B) shows quantification as a function of varying GGPP concentrations. Error bars represent mean ± 1 s.d. across 3 wells (1 of 2 trials). Scale bar, 200 µm. (TIF) [file pbio.1001379.s010.tif]

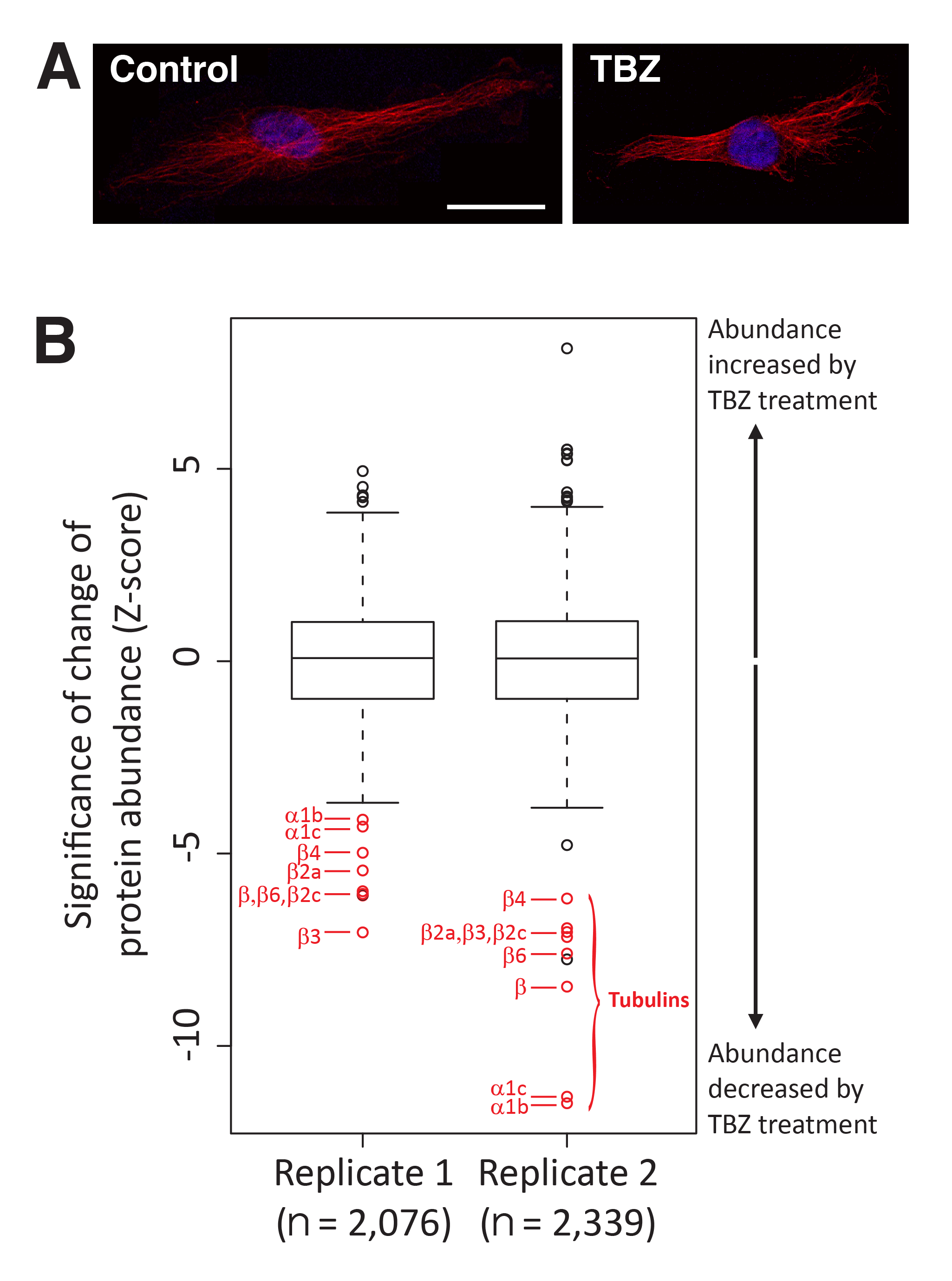

Supplement: Figure S11 — Immunohistochemical analysis of β-tubulin does not show a definite distinction between 1% DMSO-treated control and 1% DMSO, 250 µM TBZ-treated HUVECs (A), but tubulins in HUVECs identified by a quantitative mass-spectroscopy analysis were significantly reduced with TBZ treatment (B). Scale bar in (A), 20 µm. (TIF) [file pbio.1001379.s011.tif]

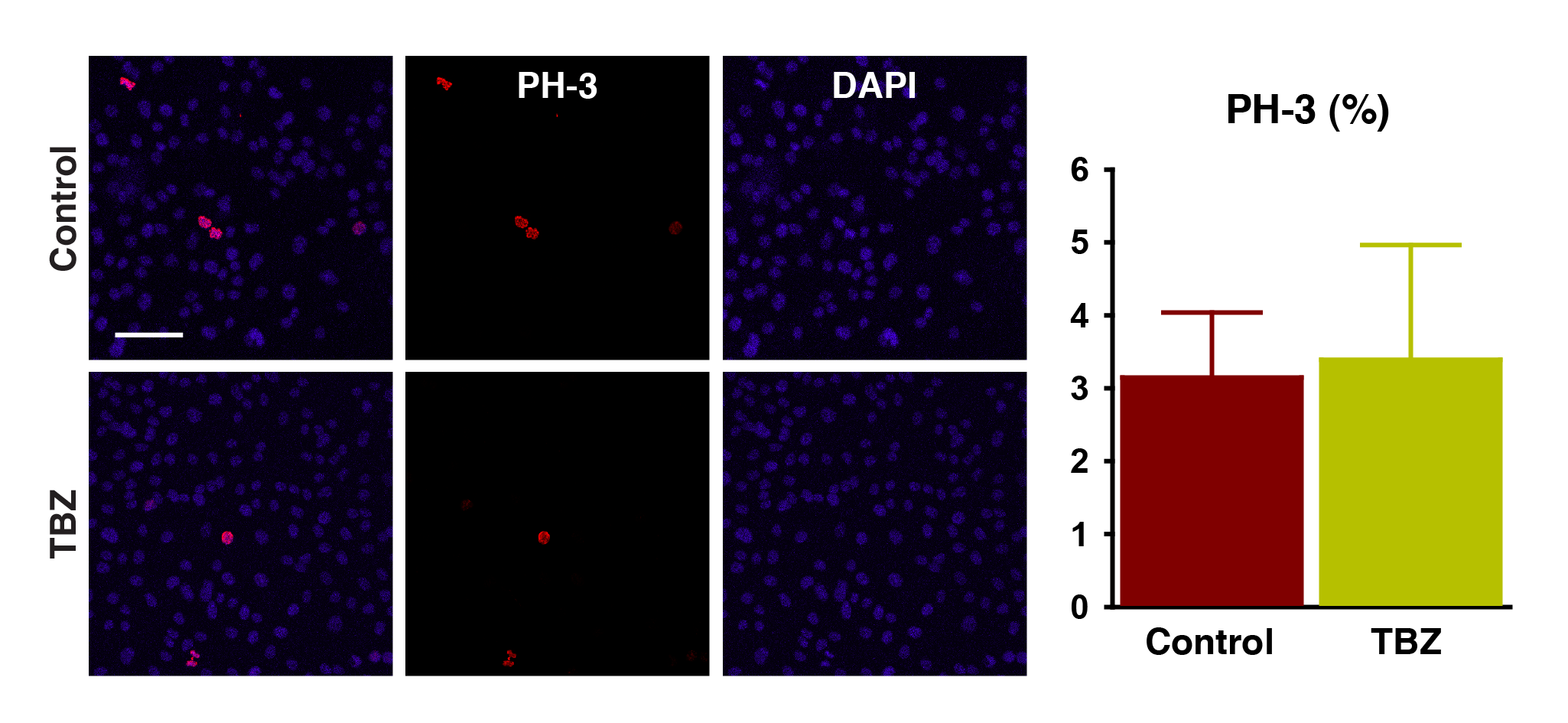

Supplement: Figure S12 — TBZ treatment does not significantly affect the proliferation of HT1080 cells. Error bars represent mean ± 1 s.d. across 14–15 fields of view of 200× magnification confocal microscopy cell images across 2 independent experiments. Scale bar, 100 µm. (TIF) [file pbio.1001379.s012.tif]

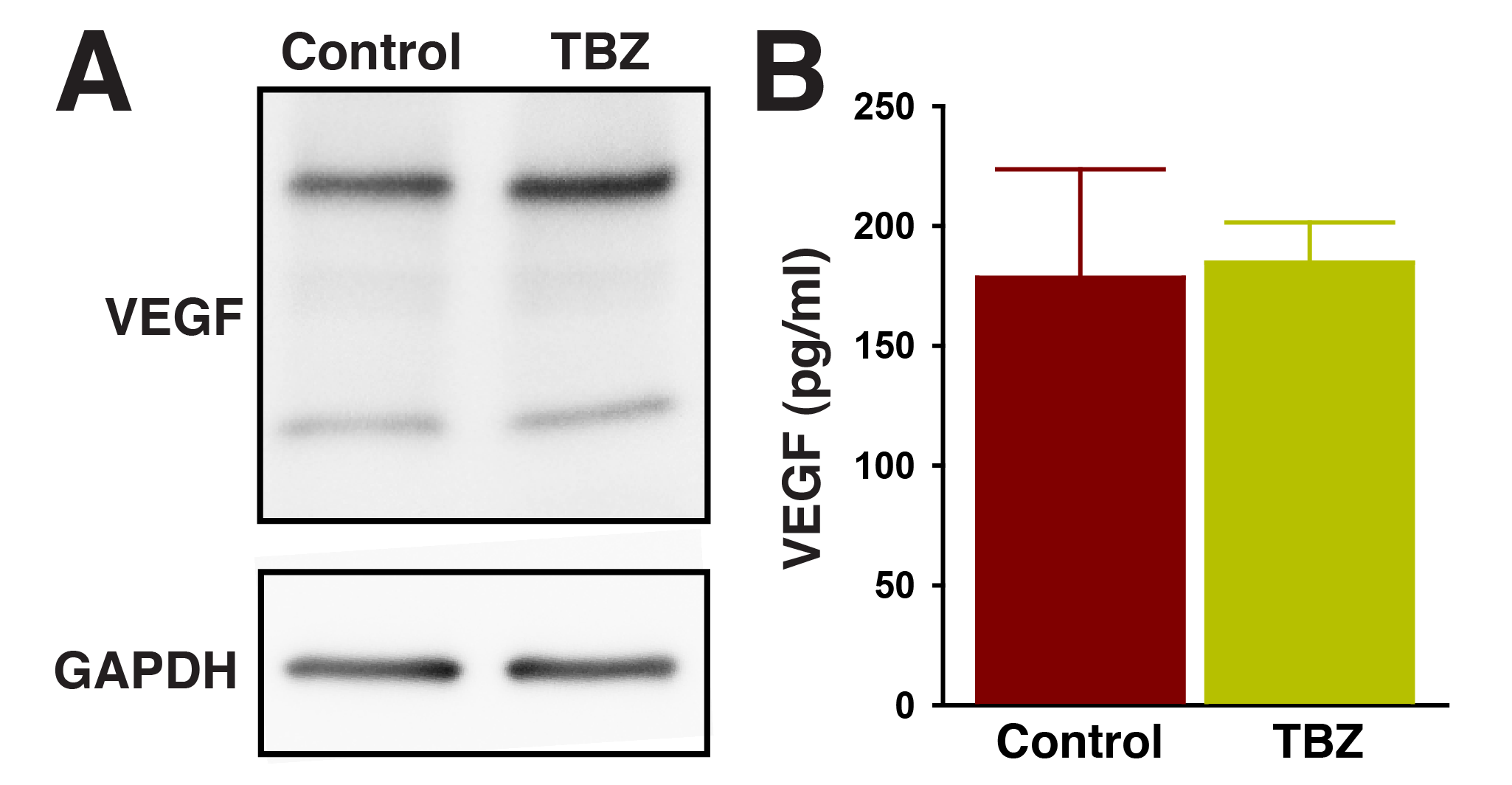

Supplement: Figure S13 — TBZ does not significantly alter intracellular or secreted VEGF levels in HT1080 cells. VEGF levels in HT1080 cells and conditioned medium were measured in 1% DMSO-treated control and 1% DMSO, 250 µM TBZ-treated HT1080 cells, assaying cellular VEGF by Western blotting (A) and secreted VEGF by ELISA (B). Glyceraldehyde-3-phosphate dehydrogenase (GAPDH) level was measured for a Western blotting control. (TIF) [file pbio.1001379.s013.tif]
