# Supplementary material for: Evolutionarily Repurposed Networks Reveal the Well-Known Antifungal Drug Thiabendazole to Be a Novel Vascular Disrupting Agent
Source: PLoS Biol. 2012 Aug 21;10(8):e1001379. doi: 10.1371/journal.pbio.1001379 (PMC3423972; doi:10.1371/journal.pbio.1001379)
Supplement: Table S1 — Conserved genes in the vertebrate angiogenesis defect/yeast lovastatin sensitivity gene module. Bold text indicates vertebrate genes whose angiogenesis roles were known or confirmed by the literature; italic text indicates genes whose roles were predicted in [1] and confirmed in frogs and HUVEC cells in [1] and Figures 2B and S1. (DOC) [file pbio.1001379.s017.doc]

**Table S1.** Conserved genes in the vertebrate angiogenesis defect/yeast lovastatin sensitivity gene module. Bold text indicates vertebrate genes whose angiogenesis roles were known or confirmed by the literature; italic text indicates genes whose roles were predicted in ref. and confirmed in frogs and HUVEC cells in ref. and **Figs. 2B**, **S1**.

| **Human gene(s)** | **Yeast gene(s)** |
| --- | --- |
| **MAPK7** | SLT2 |
| **MAP2K1** | PBS2 |
| **MAPK14** | HOG1 |
| **PPP3R1** | CNB1 |
| **PSMA (FOLH1)** | VPS70 |
| **HMGCR** | HMG1,2 |
| **SIRT1** | HST1 |
| **CSNK2A1** | CKA1 |
| *SOX13* | IXR1 |
| *RAB11B* | YPT31 |
| *HMHA1* | SAC7/BAG7 |
| *TCEA1/3* | DST1 |
| *TBL1XR1* | SIF2 |
