# Supplementary material for: Evolutionarily Repurposed Networks Reveal the Well-Known Antifungal Drug Thiabendazole to Be a Novel Vascular Disrupting Agent
Source: PLoS Biol. 2012 Aug 21;10(8):e1001379. doi: 10.1371/journal.pbio.1001379 (PMC3423972; doi:10.1371/journal.pbio.1001379)
Supplement: Table S2 — Compounds computationally prioritized as candidate angiogenesis effectors. Nineteen alternate hierarchical clustering trials were performed varying the choice of clustering algorithm and the measure of similarity between drug-gene interaction profiles (from [13]), as described in Materials and Methods, and compounds were selected by the frequency with which they occurred in the same subcluster as lovastatin. (DOC) [file pbio.1001379.s018.doc]

**Table S2.** Compounds computationally prioritized as candidate angiogenesis effectors. 19 alternate hierarchical clustering trials were performed varying the choice of clustering algorithm and the measure of similarity between drug-gene interaction profiles (from ), as described in the Materials and Methods, and compounds were selected by the frequency with which they occured in the same subcluster as lovastatin.

| **Compound** | **Known effects on angiogenesis** | **Number of hierarchical clustering trials within two branches of lovastatin** |
| --- | --- | --- |
| FeCl_4_ |  | 16 |
| Bathophenathroline disulfonate |  | 15 |
| CuSO_4_ | Activator^‡^ | 5 |
| Nitric oxide | Activator^#^ | 3 |
| Mycophenolic acid | Inhibitor^*^ | 3 |
| Thiabendazole |  | 2 |
| 5-fluorouracil | Inhibitor^†^ | 1 |
| Floxuridine |  | 1 |

^‡^A. Parke, P. Bhattacherjee, R. M. Palmer, N. R. Lazarus, *Am J Pathol* 130, 173 (1988).

^#^D. G. Duda, D. Fukumura, R. K. Jain, *Trends Mol Med* 10, 143 (2004).

^*^S. Domhan *et al*., *Mol Cancer Ther* 7, 1656 (2008).

^†^T. Browder *et al*., *Cancer Res* 60, 1878 (2000).
